# Origin of rate enhancement and asynchronicity in iminium catalyzed Diels–Alder reactions†

**DOI:** 10.1039/d0sc02901g

**Published:** 2020-07-09

**Authors:** Pascal Vermeeren, Trevor A. Hamlin, Israel Fernández, F. Matthias Bickelhaupt

**Affiliations:** Department of Theoretical Chemistry, Amsterdam Institute of Molecular and Life Sciences (AIMMS), Amsterdam Center for Multiscale Modeling (ACMM), Vrije Universiteit Amsterdam De Boelelaan 1083 1081 HV Amsterdam The Netherlands t.a.hamlin@vu.nl f.m.bickelhaupt@vu.nl; Institute for Molecules and Materials (IMM), Radboud University Heyendaalseweg 135 6525 AJ Nijmegen The Netherlands; Departamento de Química Orgánica I, Centro de Innovación en Química Avanzada (ORFEO-CINQA), Facultad de Ciencias Químicas, Universidad Complutense de Madrid 28040 Madrid Spain israel@quim.ucm.es

## Abstract

The Diels–Alder reactions between cyclopentadiene and various α,β-unsaturated aldehyde, imine, and iminium dienophiles were quantum chemically studied using a combined density functional theory and coupled-cluster theory approach. Simple iminium catalysts accelerate the Diels–Alder reactions by lowering the reaction barrier up to 20 kcal mol^−1^ compared to the parent aldehyde and imine reactions. Our detailed activation strain and Kohn–Sham molecular orbital analyses reveal that the iminium catalysts enhance the reactivity by reducing the steric (Pauli) repulsion between the diene and dienophile, which originates from both a more asynchronous reaction mode and a more significant polarization of the π-system away from the incoming diene compared to aldehyde and imine analogs. Notably, we establish that the driving force behind the asynchronicity of the herein studied Diels–Alder reactions is the relief of destabilizing steric (Pauli) repulsion and not the orbital interaction between the terminal carbon of the dienophile and the diene, which is the widely accepted rationale.

## Abstract 2

Quantum chemical activation strain analyses revealed that iminium catalysts accelerate Diels–Alder reactions by reducing the Pauli repulsion between reactants.

## Graphical abstract

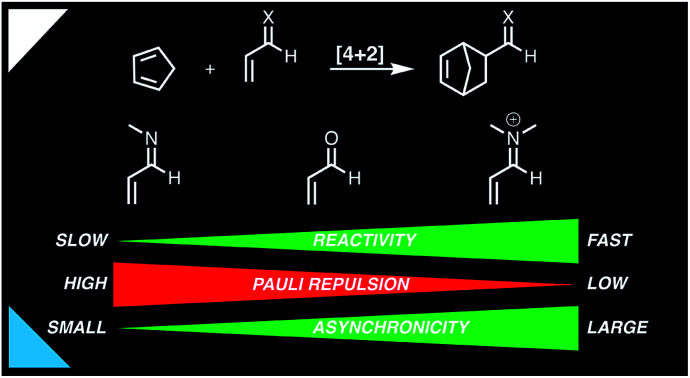

## 1. Introduction

Iminium catalysis constitutes an important branch of organocatalysis typically leading to the enantioselective β-functionalization of α,β-unsaturated aldehydes.^1^ This process is mediated by either chiral primary or secondary amine catalysts which, *via* condensation with the carbonyl compound, produce a transient iminium intermediate,^2^ which facilitates the conjugate addition to the β-carbon atom. The Knoevenagel condensation mediated by primary or secondary amines is nowadays accepted as the earliest recorded example of an iminium-catalyzed transformation.^3,4^ Since then, an impressive number of different iminium-catalyzed chemical reactions, most of them affording high enantioselectivities, have been reported.^1*b*,5,6^ For this reason, it is not surprising that this type of organocatalysis has been thoroughly applied to the synthesis of complex natural products and pharmaceuticals.^7^

The seminal report by MacMillan and co-workers in 2000 on enantioselective Diels–Alder cycloaddition reactions using iminium catalysts established the basics behind this type of organocatalysis.^8^ In analogy with Lewis acids, the term “LUMO-lowering catalysis” was coined by the authors to describe the driving force behind iminium catalysis. In the authors' own words: “…the reversible formation of iminium ions from α,β-unsaturated aldehydes and amines might emulate the equilibrium dynamics and π-orbital electronics that are inherent to Lewis acid catalysis”. Strikingly and in sharp contrast to the widely accepted rationale, we very recently demonstrated, using state-of-the-art quantum chemical calculations, that orbital interactions are not the origin of Lewis acid (LA) catalysis in Diels–Alder cycloaddition reactions.^9^ We found that although Lewis acids indeed stabilize the π-LUMO of the dienophile and, therefore, enhance the corresponding HOMO_diene_–LUMO_dienophile_ interaction,^10^ they simultaneously weaken the inverse LUMO_diene_–HOMO_dienophile_ interaction to the same extent. As a result, the total orbital interactions in both LA-catalyzed and uncatalyzed reactions are nearly identical and therefore, not responsible for the acceleration of LA-mediated reactions. Instead, the significant reduction of steric (Pauli) repulsion between the occupied orbitals of the dienophile and the diene becomes the actual driving force behind the LA-catalysis. This unprecedented electronic mechanism, which is also operative in dihalogen-catalyzed aza-Michael addition reactions^11*a*^ and related Lewis acid catalyzed (aromatic) Diels–Alder reactions,^11*b*–*d*^ contradicts the widely accepted “LUMO-lowering catalysis” as the mechanism behind the iminium catalysis.

**Scheme 1:**
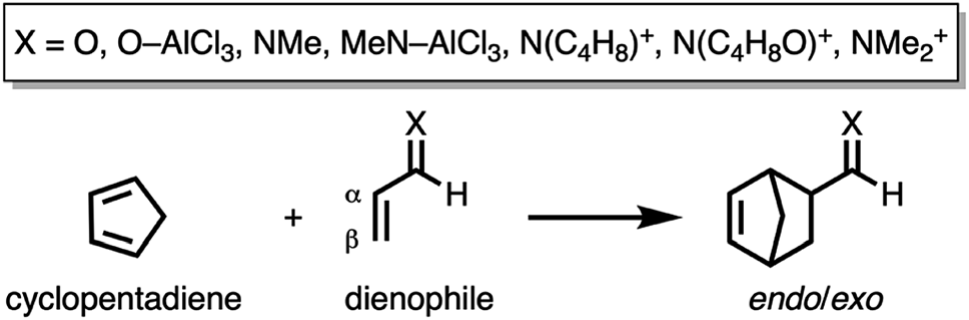
The Diels–Alder reactions between cyclopentadiene (**CP**) and various α,β-unsaturated aldehyde (**O** and **O–AlCl3**), imine (**NMe** and **MeN–AlCl3**), and iminium (**N(C4H8)+**, **N(C4H8O)+**, and **NMe2+**) dienophiles that were computationally analyzed. **N(C4H8)+** and **N(C4H8O)+** stand for the iminium ions derived from pyrrolidine and morpholine, respectively.

For this reason, herein we shall investigate the ultimate factors governing the iminium-catalysis and check the generality of our “Pauli repulsion-lowering catalysis” in this important reaction. In addition, the reasons behind the catalysis induced by the asynchronicity shall be thoroughly investigated. To this end, we have selected the Diels–Alder cycloaddition reactions involving cyclopentadiene (**CP**) and various α,β-unsaturated aldehyde (**O** and **O–AlCl3**), imine (**NMe** and **MeN–AlCl3**), and iminium (**N(C4H8)+**, **N(C4H8O)+**, and **NMe2+**) dienophiles (Scheme 1) analogous to the processes initially described by MacMillan and co-workers.^8,12^

## 2. Results and discussion

Table 1 summarizes the electronic reaction barriers (Δ*E*^‡^), reaction energies (Δ*E*_rxn_), and HOMO**CP**–LUMO_dienophile_ orbital energy gaps (Δ*ε*_H–L_) of the Diels–Alder (DA) reaction between cyclopentadiene (**CP**) and various α,β-unsaturated aldehyde (**O** and **O–AlCl3**), imine (**NMe** and **MeN–AlCl3**), and iminium (**N(C4H8)+**, **N(C4H8O)+**, and **NMe2+**) dienophiles. In all the cases, the cycloaddition reaction occurs in a concerted manner through the corresponding transition state TS (see Fig. S1 in the ESI†), after prior formation of an initial reactant complex RC which lies −3 to −10 kcal mol^−1^ below the separate reactants (the formation of this species becomes endergonic when thermal free energy corrections at 298.15 K are included, see Gibbs free energies in Table S1†).

**Table 1:** Electronic reactant complex energies (Δ*E*_RC_), reaction barriers (Δ*E*^‡^), reaction energies (Δ*E*_rxn_) (in kcal mol^−1^), and HOMO**CP**–LUMO_dienophile_ energy gaps (Δ*ε*_H–L_) (in eV) computed for the Diels–Alder reactions between cyclopentadiene (**CP**) and various α,β-unsaturated aldehyde, imine, and iminium dienophiles^a^^,^^b^

X		Δ*E*_RC_	Δ*E*^‡^	Δ*E*^‡^^c^	Δ*E*_rxn_	Δ*ε*_H–L_
**O**	*endo*	−3.6	15.2	14.3	−20.3	−6.67
	*exo*	−2.9	15.8	14.8	−20.1	
**O–AlCl3**	*endo*	−7.3	1.9	3.2	−22.6	−4.58
	*exo*	−5.8	2.4	3.3	−22.0	
**NMe**	*endo*	−3.1	18.6	17.1	−19.3	−7.37
	*exo*	−2.4	19.1	17.6	−18.9	
**MeN–AlCl3**	*endo*	−5.1	10.9	10.5	−20.7	−5.52
	*exo*	−4.5	10.4	10.4	−21.8	
**N(C4H8)+** d	*endo*	−9.1	−0.3	0.7	−23.6	−1.59
	*exo*	−8.2	−0.1	1.1	−22.7	
**N(C4H8O)+** d	*endo*	−9.3	−1.4	−0.1	−24.1	−1.51
	*exo*	−8.4	−0.1	1.1	−22.7	
**NMe2+**	*endo*	−9.7	−2.0	−0.6	−24.1	−1.38
	*exo*	−8.7	−1.8	−0.4	−23.0	

a All energies were computed at M06-2X/def2-TZVP and were referenced to the isolated reactants.

b See ESI Table S1 for Gibbs free reaction barriers and energies.

c Computed at DLPNO-CCSD(T)/def2-QZVPP//M06-2X/def2-TZVP.

d **N(C4H8)+** and **N(C4H8O)+** stand for the iminium ions derived from pyrrolidine and morpholine, respectively.

Three distinct trends can be observed. In the first place, for a given substrate, the *endo* DA reaction proceeds with a 0.5–1.5 kcal mol^−1^ lower reaction barrier than the *exo* DA reaction in line with our previous theoretical studies and experimentally observed product ratios.^12,13^ There is, however, one exception, namely, the Diels–Alder reaction between **CP** and **MeN–AlCl3**. For this reaction, the *exo* pathway has a slightly lower barrier than the *endo* pathway. Secondly, both the uncatalyzed and LA-catalyzed DA reactions involving an α,β-unsaturated imine as the dienophile have comparatively higher reaction barriers, 18.6 and 10.9 kcal mol^−1^, for **NMe** and **MeN–AlCl3**, respectively (*endo* approach), than the corresponding aldehyde analogs, 15.2 and 1.9 kcal mol^−1^, for **O** and **O–AlCl3**, respectively. Thirdly, introducing an iminium catalyst significantly accelerates the DA reaction by lowering the reaction barrier from 18.6 kcal mol^−1^ for **NMe** to −2.0 kcal mol^−1^ for **NMe2+**. This acceleration is even higher than that caused by the strong Lewis acid catalyst AlCl_3_. Similar values were found for the iminium dienophiles derived from pyrrolidine and morpholine: **N(C4H8)+** and **N(C4H8O)+**, respectively. Furthermore, there is a good linear correlation (*R*^2^ = 0.91) between the reaction barrier (Δ*E*^‡^) and the HOMO**CP**–LUMO_dienophile_ orbital energy gap (Δ*ε*_H–L_, see Fig. S2 in the ESI†). Thus, one might, indeed, suspect that lower and more favorable reaction barriers directly arise due to a more stabilizing orbital interaction as a result of priorly reported lowering of the LUMO_dienophile_.^10^ This result seemingly confirms that this commonly accepted textbook explanation could be the decisive factor behind the computed reactivity trends. We will show next that, similar to the situation for Lewis-acid catalysis,^9^ this is not the case in iminium-ion catalysis either.

**Fig. 1:**
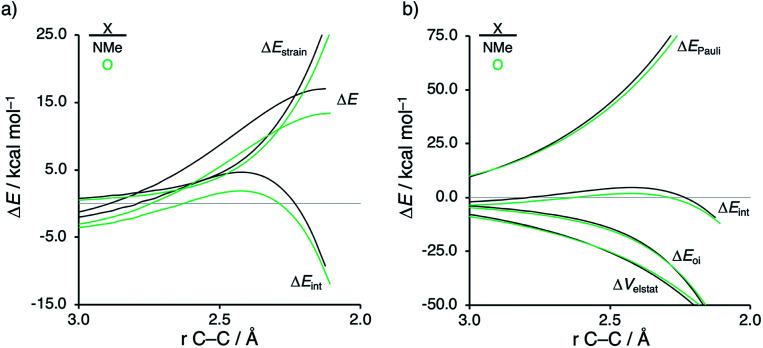
(a) Activation strain analyses and (b) energy decomposition analyses of the Diels–Alder reactions between the **CP** and **O** and **NMe** going from the reactants to the transition states, where the energy values are projected onto the shorter newly forming C**CP**⋯C_β_ bond between **CP** and the dienophile, computed at ZORA-M06-2X/TZ2P//M06-2X/def2-TZVP.

First, we aim to gain quantitative insight into the physical factors leading to the computed difference in reactivity between the uncatalyzed *endo* Diels–Alder reactions involving the parent α,β-unsaturated aldehyde and imine dienophiles by applying the activation strain model (ASM) of reactivity.^14^ This model, which is also known as the distortion/interaction model,^14*d*^ involves decomposing the electronic energy (Δ*E*) into two distinct energy terms, namely, the strain energy (Δ*E*_strain_) that results from the deformation of the individual reactants and the interaction energy (Δ*E*_int_) between the deformed reactants along the reaction coordinate, defined, in this case, by the shorter newly forming C**CP**⋯C_β_ bond between **CP** and the dienophile. This critical reaction coordinate undergoes a well-defined change throughout the reaction and has successfully been used in the past for the analysis of similar reactions.^9,15,16^Fig. 1a shows the activation strain diagrams (ASDs) from the reactants to the transition states (see Fig. S5† for the complete reaction profiles) for the Diels–Alder reactions between **CP** and the dienophiles **O** and **NMe**. The enhanced reactivity for the reaction of **O** originates from both a less destabilizing strain energy and a more stabilizing interaction energy (rather similar ASDs were obtained using the BP86-D3 and B3LYP-D3 functionals, therefore supporting the selected computational methodology for the present study, see Fig. S6–S9 in the ESI†).

By inspecting and comparing the DA reaction modes of **O** and **NMe**, we can ascribe their differences in Δ*E*_strain_ to the higher degree of asynchronicity of **O** (**O**: Δ*r*^TS^_C⋯C_ = 0.19 Å; and **NMe**: Δ*r*^TS^_C⋯C_ = 0.14 Å, where Δ*r*^TS^_C⋯C_ is the difference between the newly forming C⋯C bond lengths in the TS), which leads to a lower degree of deformation of the reactants since the C**CP**⋯C_β_ bond forms ahead of the C**CP**⋯C_α_ bond (see Fig. S1† for transition state structures).^17^ Later on, we analyze and explain the origin of, and differences between, the degrees of asynchronicity of the herein studied Diels–Alder reactions. In addition to the strain, the important role of the interaction energy in the observed reactivity trend prompted the analysis of the different contributors to the interaction energy using the canonical energy decomposition analysis (EDA).^18^ Our canonical EDA decomposed the Δ*E*_int_ between the reactants into three physically meaningful energy terms: classical electrostatic interaction (Δ*V*_elstat_), steric (Pauli) repulsion (Δ*E*_Pauli_) which, in general, arises from the two-center four-electron repulsion between the closed-shell orbitals of both reactants, and stabilizing orbital interactions (Δ*E*_oi_) that account, among others, for HOMO–LUMO interactions. The corresponding energy decomposition analysis (EDA) results for the Diels–Alder reactions of **O** and **NMe** are presented in Fig. 1b. The differences in Δ*E*_int_ between **O** and **NMe** can solely be assigned to the Pauli repulsion. The electrostatic and orbital interactions are, on the other hand, similar or even more stabilizing for **NMe** compared to **O**, despite the more favorable HOMO**CP**–LUMO_dienophile_ gap (Δ*ε*_H–L_, see Table 1) computed for the latter system.

**Fig. 2:**
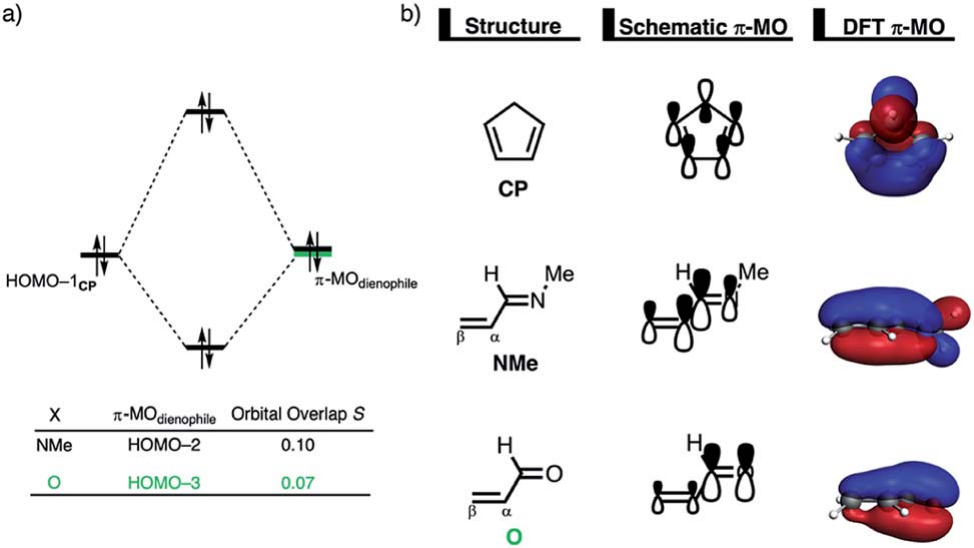
(a) Molecular orbital diagram and the most significant occupied orbital overlaps of the Diels–Alder reactions between **CP** and dienophiles **NMe** and **O** and (b) key occupied orbitals (isovalue = 0.03 au^−3/2^) computed at consistent geometries with a C**CP**⋯C_β_ bond length between **CP** and the dienophile of 2.125 Å at ZORA-M06-2X/TZ2P//M06-2X/def2-TZVP.

The origin of the less destabilizing Pauli repulsion for the Diels–Alder reaction involving **O** was investigated by performing a Kohn–Sham molecular orbital (KS-MO) analysis.^18*b*,19^ The occupied molecular orbitals of **CP**, as well as, **O** and **NMe** were quantified at consistent geometries with a C**CP**⋯C_β_ bond length between **CP** and the dienophile of 2.125 Å (Fig. 2a). Performing this analysis at a consistent point along the reaction coordinate (near all transition structures), rather than the transition state alone, ensures that the results are not skewed by the position of the transition state.^14,20^ The most important occupied π-MO of the dienophile involved in the two-center four-electron interaction are the HOMO−2 and HOMO−3 of **NMe** and **O**, respectively, where all 2p_π_ AOs are in-phase. The contributing occupied orbital of **CP** is HOMO−1, where all 2p_π_ AOs located on both reacting C=C double bonds are in-phase. The orbital overlap between the HOMO−1**CP** and the occupied π-MO of the dienophile is larger (*S* = 0.10) and, therefore, more destabilizing for **NMe** and smaller and less destabilizing for **O** (*S* = 0.07). The difference in the electronegativity of the heteroatoms is the reason behind the decreased occupied–occupied orbital overlap. The oxygen of **O** is more electronegative than the nitrogen of **NMe** and, therefore, polarizes more π-electron density away from the terminal carbon of the C=C double bond of the dienophile, which is directly involved in the Diels–Alder reaction.

Inspection of the spatial distribution of the occupied π-MO of the dienophile (Fig. 2b) reveals that the HOMO−3 of **O** is polarized towards the oxygen, which results in a small orbital amplitude on the reactive C=C bond, while in the case of **NMe** this polarizing effect is minimal. Thus, it can be concluded that a large difference in electronegativity induces a significant reduction of the electron density at the reactive C=C double bond of the dienophile which results in a lower 〈HOMO−1**CP**|HOMO−3**O**〉 overlap and ultimately, in a less destabilizing Pauli repulsion and a lower reaction barrier. We have, as priorly discussed, observed this exact phenomenon in our analysis of Lewis acid-catalyzed Diels–Alder and aza-Michael addition reactions.^9,11^ This demonstrates that the applicability of the concept of catalysis through reduced steric (Pauli) repulsion, caused by polarizing the filled π-orbitals on the C=C double bond away from the incoming reactant, is general and not limited only to Lewis acid-catalyzed organic reactions.

**Fig. 3:**
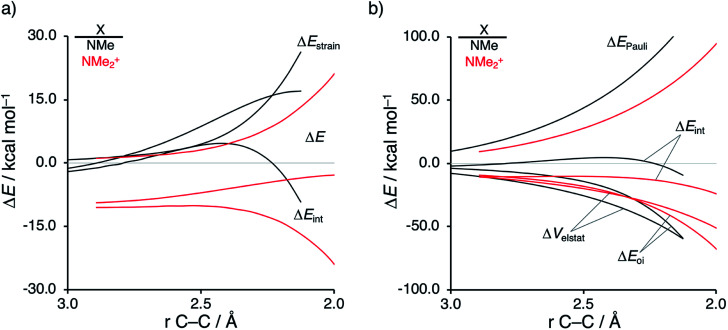
(a) Activation strain analyses and (b) energy decomposition analyses of the Diels–Alder reactions between the **CP** and **NMe** and **NMe2+** going from the reactants to the transition states, where the energy values are projected onto the shorter newly forming C**CP**⋯C_β_ bond between **CP** and the dienophile, computed at ZORA-M06-2X/TZ2P//M06-2X/def2-TZVP.

Next, we want to understand the driving mechanism behind iminium-catalyzed Diels–Alder reactions, *i.e.*, why does the Diels–Alder reaction with α,β-unsaturated iminium dienophiles have markedly lower reaction barriers than their imine analogs. Fig. 3 shows the activation strain diagram (ASD) from the reactants to the transition states for the Diels–Alder reactions between **CP** and the dienophiles **NMe** and **NMe2+**.^14*e*^ The accelerated reactivity of the iminium catalyst (**NMe2+**) originates from both a less destabilizing strain energy, as well as a much more stabilizing interaction energy (Fig. 3a). Diels–Alder reactions with more commonly employed iminium catalysts, *i.e.*, pyrrolidine (**N(C4H8)+**) and morpholine (**N(C4H8O)+**), exhibit identical reactivity trends to **NMe2+** and are provided in the ESI (see Fig. S10†).

The difference in strain energy can again be explained by looking at the degree of asynchronicity, which is the largest for the iminium dienophile (**NMe**: Δ*r*^TS^_C⋯C_ = 0.14 Å, *S*_*y*_ = 0.93; **NMe2+**: Δ*r*^TS^_C⋯C_ = 0.86 Å, and *S*_*y*_ = 0.77, where Δ*r*^TS^_C⋯C_ is the difference in newly forming C⋯C bond lengths in the TS and *S*_*y*_ stands for the computed synchronicity^16^). The higher degree of asynchronicity of **NMe2+** leads to a lower degree of deformation of the reactants since the C_β_⋯C**CP** bond forms before the C_α_⋯C**CP** bond. Note that, in the product, the strain energies of both **NMe** and **NMe2+** are identical because both new C⋯C bonds are now completely formed and the reactants, therefore, are deformed to the same degree (see Fig. S10† for the complete reaction profiles). To understand why **NMe2+** goes with a more stabilizing interaction energy compared to **NMe**, we applied the energy decomposition analysis (EDA) (Fig. 3b). In contrast to the commonly accepted view that iminium catalysts enhance the orbital interactions of the Diels–Alder reactions,^8,10^ we find that the difference in Δ*E*_Pauli_ curves exclusively determines the more stabilizing Δ*E*_int_ for **NMe2+** and, thus, contributes to the lowering of the reaction barrier. In contrast, the Δ*V*_elstat_ and Δ*E*_oi_ terms, on the other hand, are, in the transition state region, more stabilizing for the uncatalyzed Diels–Alder reaction with **NMe**, even though the **NMe2+** system benefits from a much more favorable HOMO**CP**–LUMO_dienophile_ energy gap (see Table 1).

**Fig. 4:**
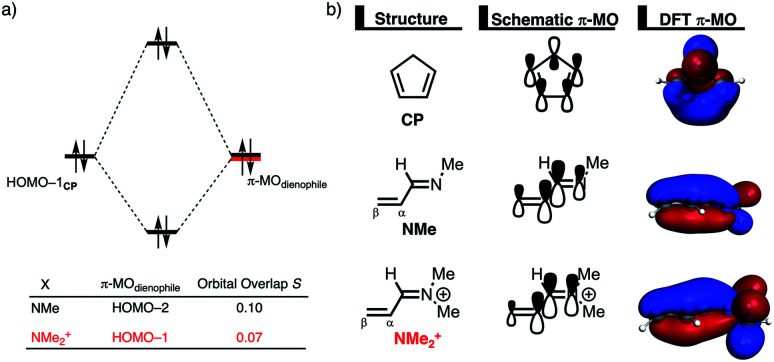
Molecular orbital diagrams of the key orbital interactions of the Diels–Alder reactions between **CP** and **NMe** and **NMe2+**: (a) most significant occupied orbital overlaps and (b) 3D plots of the involved occupied orbitals (isovalue = 0.03 au^−3/2^). All data were computed at consistent geometries with a C**CP**⋯C_β_ bond length between **CP** and the dienophile of 2.125 Å at ZORA-M06-2X/TZ2P//M06-2X/def2-TZVP.

The less destabilizing Pauli repulsion for the reaction involving **NMe2+** originates from a reduced occupied–occupied orbital overlap with incoming **CP**. The most important occupied π-MO of the dienophile participating in the two-center four-electron interaction are the HOMO−2 and HOMO−1 of **NMe** and **NMe2+**, respectively, where all 2p_π_ AOs are in-phase. Furthermore, the contributing occupied orbital of **CP** is HOMO−1, where all 2p_π_ AOs located on both reacting C=C double bonds are in-phase. The HOMO–HOMO overlap lowers from 0.10 for **NMe** to 0.07 for **NMe2+**, which is in line with the trend in Pauli repulsion (Fig. 4a). The difference in the orbital overlap between **NMe** and **NMe2+** is a direct consequence of their difference in asynchronicity. For a more asynchronous reaction (**NMe2+**), the reactants have almost exclusively orbital overlap on the β-carbon side of the dienophile, and, therefore, less destabilizing Pauli repulsion and a lower reaction barrier. Besides being more asynchronous, the HOMO−2 of **NMe2+** has lower orbital amplitude on the β-carbon compared to **NMe**, which, in turn, also leads to the computed lower occupied–occupied orbital overlap (Fig. 4b).

**Fig. 5:**
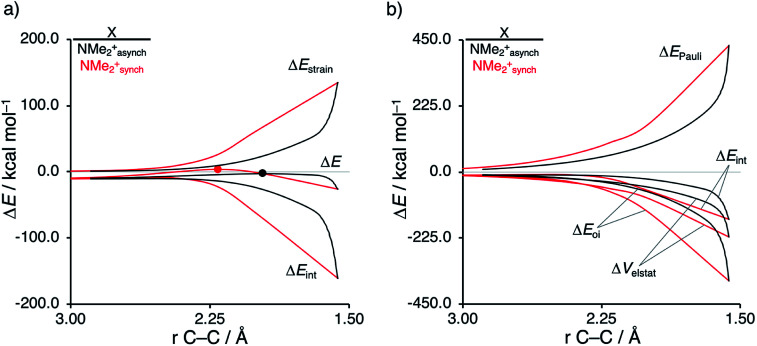
(a) Activation strain analyses and (b) energy decomposition analyses of the asynchronous (black) and constrained synchronous (red) Diels–Alder reactions between **CP** and **NMe2+** going from the reactants to the product, where the transition states are indicated with a dot and the energy values are projected onto the shorter newly forming C**CP**⋯C_β_ bond between **CP** and **NMe2+**, computed at ZORA-M06-2X/TZ2P//M06-2X/def2-TZVP for **NMe2+asynch** and ZORA-M06-2X/TZ2P for **NMe2+synch**.

As described above, asynchronicity is a key factor in these reactions. Indeed, a very good linear relationship (correlation coefficient *R*^2^ of 0.96) was found when plotting the computed activation barriers *versus* the corresponding difference in newly forming C⋯C bond lengths in the TS, Δ*r*^TS^_C⋯C_ (see Fig. S11 in the ESI†). The origin of the asynchronicity of the Diels–Alder reactions that significantly contributes to the intrinsic catalytic effect of iminium catalysis (by allowing for less reactant deformation and strain; *vide supra*), deserves further analysis. To this end, we compared the actual concerted asynchronous Diels–Alder reaction to the analogous process which is artificially constrained to be concerted synchronous. In Fig. 5, we solely focus on the activation strain diagrams (ASDs) of the asynchronous and synchronous **NMe2+** DA reaction for which the effects are the largest. The ASDs of **NMe** and the more realistic iminium **N(C4H8O)+** possess the same, albeit with less pronounced features (see Fig. S12 and S13†). The synchronous DA reaction proceeds with a higher barrier compared to its asynchronous counterpart (ΔΔ*E*^‡^ = 5.7 kcal mol^−1^), even though the synchronous DA reaction has surprisingly more stabilizing interaction energy. The strain energy is initially the largest for the synchronous DA reaction because both newly forming C⋯C bonds between **CP** and **NMe2+** are formed simultaneously, causing all involved carbon atoms to pyrimidalize at the same time (*i.e.*, more deformation and thus more strain). However, on the product side (right side of ASD), the strain energies of both the asynchronous and synchronous DA reactions are identical, because, in both cases, the reactants end up in identical products and are, thus, deformed to the same extent.

Next, we turn to the EDA to get a more detailed insight into the counterintuitive finding that the interaction energy is more stabilizing for the synchronous DA reaction. In contrast with the current view that the asynchronicity originates from enhanced orbital interactions,^21^ we found that the significantly larger Pauli repulsion for the synchronous DA reaction compared to the asynchronous DA reaction constitutes the actual driving force behind the asynchronous reaction mode. In order to relieve the highly destabilizing Pauli repulsion originating from a larger occupied–occupied orbital overlap (see Fig. S14†) of the synchronous DA reaction, the reaction mode becomes asynchronous despite this resulting in a loss of the stabilizing orbital and electrostatic interactions. The delicate interplay between this reduction of unfavorable Pauli repulsion and loss of favorable orbital and electrostatic interactions determines the degree of asynchronicity. Thus, Diels–Alder reactions only become asynchronous when the gain in stability, as a response to the reduced Pauli repulsion, is large enough to compensate for the significant loss of stabilizing interactions, that is, when the catalyst induces sufficient asymmetry in the occupied π-MOs of the dienophile. We do, as previously reported in the literature,^20^ find a larger 2p_*z*_-coefficient on the β-carbon of the **NMe2+** LUMO than on the α-carbon (Fig. S15†). But, this does not lead to more stabilizing orbital interactions for the asynchronous DA reaction, because the orbital overlap of both the normal electron demand, 
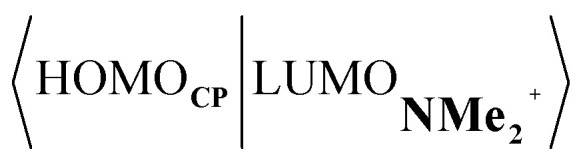
, and inverse electron demand, 
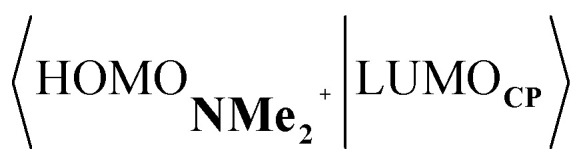
, is, along the entire reaction coordinate, larger for, and therefore also favorable for, the synchronous compared to the asynchronous reaction mode (see Fig. S16†).

**Fig. 6:**
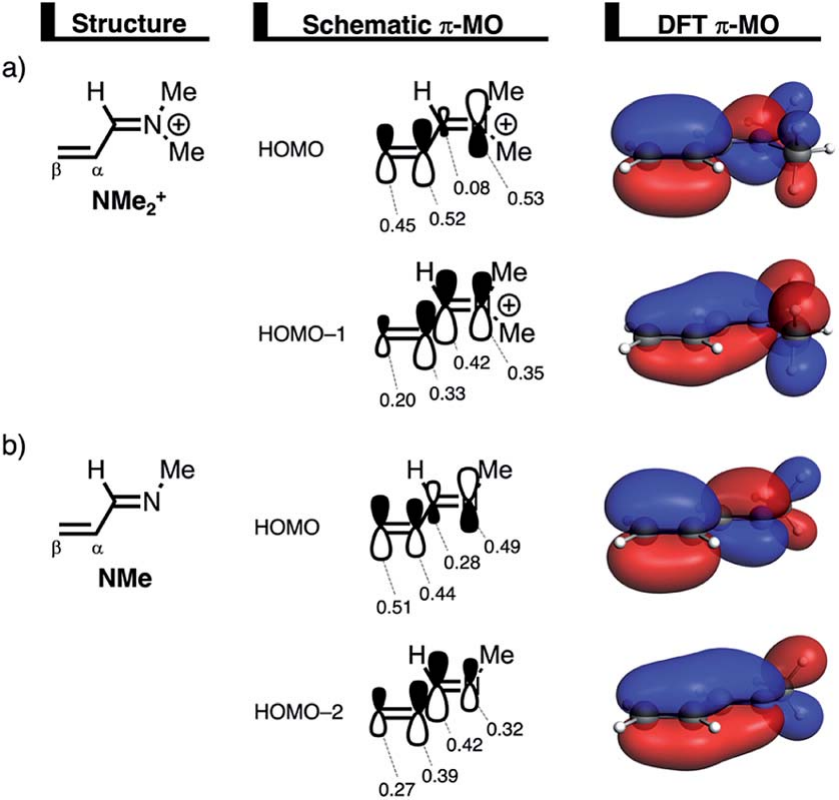
Key occupied π-MOs (isovalue = 0.03 au^−3/2^) computed at the equilibrium structures of (a) **NMe2+** and (b) **NMe**, where the MO-coefficients of the carbon and nitrogen 2p_*z*_ atomic orbitals, contributing to the occupied orbitals, are shown in the schematic π-MOs.

We can trace the larger orbital overlap and, consequently, stronger Pauli repulsion for the synchronous DA reaction back to the orbital amplitude of the key occupied orbitals, HOMO and HOMO−1, of **NMe2+** on the α- and β-carbon atoms (Fig. 6a). The larger MO-coefficient of the 2p_*z*_ atomic orbital on the α-carbon of the dienophile leads to a larger orbital overlap and, therefore, more Pauli repulsion with the filled orbitals of **CP** than the β-carbon, which has a smaller MO-coefficient and, as a consequence, less orbital overlap and Pauli repulsion with **CP**. To reduce the larger Pauli repulsion originating from the α-carbon of **NMe2+** and **CP**, the newly forming C_α_⋯C**CP** bond must be elongated to a larger extent than the analogous bond formed between C_β_⋯C**CP**, making the DA reaction asynchronous.

In addition, we want to understand why the DA reaction involving **NMe2+** is significantly more asynchronous than **NMe** (**NMe2+**: Δ*r*^TS^_C⋯C_ = 0.86 Å; **NMe**: Δ*r*^TS^_C⋯C_ = 0.14 Å). In order to understand this difference, we need to compare the MO-coefficients on the α- and β-carbon of the key HOMOs of **NMe2+** and **NMe**, Fig. 6a and b, respectively. As priorly discussed, both key occupied orbitals of **NMe2+** have a larger MO-coefficient on the α-than on the β-carbon and, thus, both work in favor of an asynchronous reaction mode. The MO-coefficients of the key HOMOs of **NMe**, on the other hand, do not both point towards the observed asynchronous reaction. As expected, the MO-coefficient of the α-carbon of HOMO−2 is larger than that of the β-carbon, driving the reaction to the observed asynchronous reaction mode. This effect, however, gets partly, but not completely, countered by the MO-coefficients of the HOMO of **NMe**, which has a larger orbital amplitude on the β-carbon than on the α-carbon, resulting in a DA reaction which has a smaller degree of asynchronicity than **NMe2+**.

**Fig. 7:**
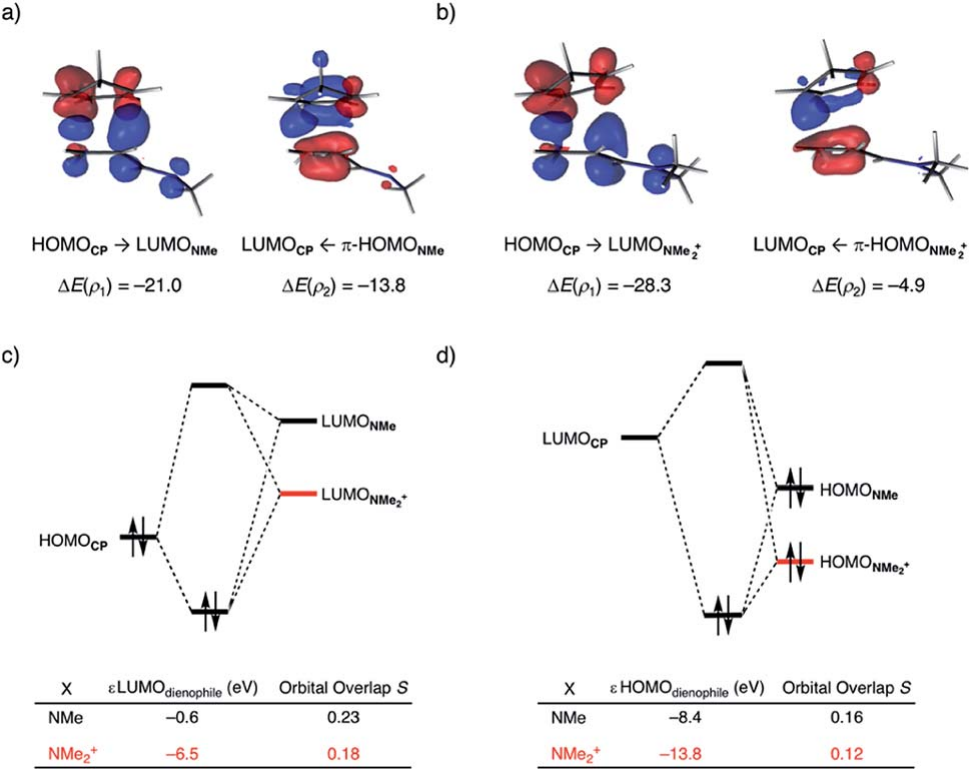
NOCV deformation densities Δ*ρ* (isovalue = 0.0015 au) and associated energies Δ*E*(*ρ*) (in kcal mol^−1^) for the normal electron demand (NED), HOMO**CP**–LUMO_dienophile_, and inverse electron demand (IED), LUMO_dienophile_–HOMO**CP**, where the color flow is red → blue, for (a) **NMe**, (b) **NMe2+**; the Kohn–Sham molecular orbital analysis for (c) NED, and (d) IED. All data were computed at consistent geometries with a C**CP**⋯C_β_ bond length between **CP** and the dienophile of 2.125 Å at ZORA-M06-2X/TZ2P//M06-2X/def2-TZVP.

At last, we address why the current rationale behind iminium-catalyzed Diels–Alder reactions is misleading, and thus, why the orbital interactions for **NMe2+** are less stabilizing than for **NMe** even though the former system exhibits a smaller HOMO**CP**–LUMO_dienophile_ energy gap by applying the natural orbitals for chemical valence (NOCV) extension of the EDA.^22^ This method confirms that although the normal electron demand (NED) interaction, between HOMO**CP**–LUMO_dienophile_, is enhanced in the **NMe2+** reaction (ΔΔ*E*(*ρ*_1_) = −7.3 kcal mol^−1^), the inverse electron demand (IED) interaction, between the LUMO**CP** and HOMO_dienophile_, is significantly weakened in the **NMe2+** system (ΔΔ*E*(*ρ*_2_) = 8.9 kcal mol^−1^). As a result, the total orbital interactions are less stabilizing in the catalyzed reaction (Fig. 7a and b). The mechanism behind these EDA-NOCV results is found in the following. In line with the original rationale behind iminium-catalyzed DA reactions,^8,10^ the iminium catalyst stabilizes the LUMO_dienophile_ from −0.6 eV for **NMe** to −6.4 eV for **NMe2+**, leading to a smaller 
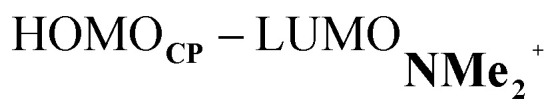
 energy gap compared to that of the **NMe** analog (Fig. 7c). This effect surpasses the unfavorable reduction of orbital overlap, which finds its origin in the priorly discussed increased asynchronicity, and, therefore, enhances the NED interaction. The iminium catalyst, however, stabilizes all **NMe2+** orbitals, thus also the HOMO_dienophile_ from −8.4 eV for **NMe** to −13.8 eV **NMe2+**, which, in turn, results in a larger 
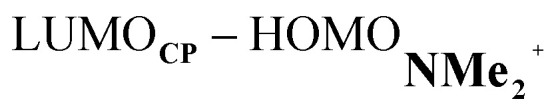
 gap and, together with a less favorable orbital overlap, weakens the IED interaction (Fig. 7d). The weakening of the IED interaction effectively overrules the more stabilizing NED interaction and, for this reason, the total orbital interactions of **NMe2+** are less stabilizing than for **NMe**.

## 3. Conclusions

Our computational study, based on the activation strain model and canonical energy decomposition analysis, reveals that iminium groups (**NMe2+**) efficiently catalyze the Diels–Alder reaction between cyclopentadiene (**CP**) and α,β-unsaturated dienophiles by accelerating the reaction by up to 15 orders of magnitude compared to the uncatalyzed reactions. Furthermore, we found that the uncatalyzed reactions involving α,β-unsaturated aldehyde dienophiles (**O**) proceed with a consistently lower reaction barrier than the imine (**NMe**) analogs.

Strikingly, the enhanced reactivity of the iminium-catalyzed Diels–Alder reactions is exclusively caused by a markedly diminished two-center four-electron steric (Pauli) repulsion between the π-systems of **CP** and **NMe2+** and not from enhanced orbital interactions as a response to the lowering of the LUMO_dienophile_. In fact, the net orbital interactions in the iminium reaction are even less stabilizing because of a weakening of the IED HOMO_dienophile_–LUMO_diene_ interaction. This finding contradicts the widely accepted LUMO-lowering catalysis as the actual electronic mechanism behind this mode of catalysis.

Most importantly, the present study establishes for the first time and in a quantitative manner the causal relationship between, on the one hand, synchronicity and reactivity in Diels–Alder cycloaddition reactions and, on the other hand, the Pauli repulsive occupied–occupied orbital overlap between the reactants and the way it depends on the shape of the occupied π-MO of the dienophile.

The reason for the Pauli repulsion lowering-catalysis is that the occupied π-orbitals of the dienophile have a larger orbital amplitude on the α-compared to the β-carbon, resulting in less occupied–occupied orbital overlap between **CP** and the β-carbon than the α-carbon of the dienophile. This asymmetry introduces a bias towards forming the C**CP**⋯C_β_ bond ahead of the C**CP**⋯C_α_ bond and results in a highly asynchronous reaction. This circumstance has two stabilizing and thus barrier-lowering consequences: (i) reduced Pauli repulsive occupied–occupied overlap and thus a more stabilizing interaction between reactants in the TS at the expense of a less significant loss in bonding HOMO–LUMO overlap and thus stabilizing orbital interaction; and (ii) less pressure on the reactants to deform and thus a less destabilizing activation strain in the TS.

## Conflicts of interest

There are no conflicts to declare.

## Supplementary Material

SC-011-D0SC02901G-s001
