# Supplementary material for: Origin of rate enhancement and asynchronicity in iminium catalyzed Diels–Alder reactions
Source: Chem Sci. 2020 Jul 9;11(31):8105–12. doi: 10.1039/d0sc02901g (PMC8163289; doi:10.1039/d0sc02901g)
Supplement: SC-011-D0SC02901G-s001 [file SC-011-D0SC02901G-s001.pdf]

Electronic Supplementary Material (ESI) for Chemical Science.
This journal is © The Royal Society of Chemistry 2020

Contents

Computational Details

Table S1. Gibbs free reactant complexes (ΔG_{RC}), reaction barriers ($\Delta G^{‡}$), reaction energies (ΔG_{rxn}) (in kcal mol^{-1}), computed for the Diels-Alder reactions between cyclopentadiene (**CP**) and various aldehyde, imine, and iminium dienophiles.

Figure S1. Transition state structures for the Diels–Alder reaction between cyclopentadiene and various dienophiles forming an endo (left) or exo (right) cycloadduct, computed at M06-2X/def2-TZVP.

Figure S2: Activation barriers for the Diels-Alder reactions versus the reactants' $HOMO_{\mathbf{CP}}$–$LUMO_{dienophile}$ gaps ($\Delta\varepsilon$), computed at M06-2X/def2-TZVP.

Figure S3 a) Activation strain analyses and b) energy decomposition analyses of the Diels-Alder reactions between the **CP** and **O** and **NMe** going from the reactants to the transition states, where the energy values are projected onto the stretch of the C_{α}•••C_{β} bond of the dienophile, computed at ZORA-M06-2X/TZ2P//M06-2X/def2-TZVP.

Figure S4. a) Activation strain analyses and b) energy decomposition analyses of the Diels-Alder reactions between the **CP** and **NMe** and $\mathbf{NMe_2}^+$ going from the reactants to the transition states, where the energy values are projected onto the stretch of the C_{α}•••C_{β} bond of the dienophile, computed at ZORA-M06-2X/TZ2P//M06-2X/def2-TZVP.

Figure S5. a) Activation strain analyses and b) energy decomposition analyses of the Diels-Alder reactions between the **CP** and **O** and **NMe** going from the reactants to the products, where the energy values are projected onto the shorter newly forming $C_{\mathbf{CP}}$•••C_{β} bond between **CP** and the dienophile, computed at ZORA-M06-2X/TZ2P//M06-2X/def2-TZVP.

Figure S6. a) Activation strain analyses and b) energy decomposition analyses of the Diels-Alder reactions between the **CP** and **O** and **NMe** going from the reactants to the transition states, where the energy values are projected onto the shorter newly forming $C_{\mathbf{CP}}$•••C_{β} bond between the **CP** and the dienophile, computed at ZORA-BP86-D3(BJ)/TZ2P//M06-2X/def2-TZVP.

Figure S7. a) Activation strain analyses and b) energy decomposition analyses of the Diels-Alder reactions between the **CP** and **NMe** and $\mathbf{NMe_2}^+$ going from the reactants to the transition states, where the energy values are projected onto the shorter newly forming $C_{\mathbf{CP}}$••• C_{β} bond between **CP** and the dienophile, computed at ZORA-BP86-D3(BJ)/TZ2P//M06-2X/def2-TZVP.

Figure S8. a) Activation strain analyses and b) energy decomposition analyses of the Diels-Alder reactions between the **CP** and **O** and **NMe** going from the reactants to the transition states, where the energy values are projected onto the shorter newly forming $C_{\mathbf{CP}}$•••C_{β} bond between the **CP** and the dienophile, computed at ZORA-B3LYP-D3(BJ)/TZ2P//M06-2X/def2-TZVP.

Figure S9. a) Activation strain analyses and b) energy decomposition analyses of the Diels-Alder reactions between the **CP** and **NMe** and $\mathbf{NMe_2}^+$ going from the reactants to the transition states, where the energy values are projected onto the shorter newly forming $C_{\mathbf{CP}}$•••C_β bond between **CP** and the dienophile, computed at ZORA-BP86-D3(BJ)/TZ2P//M06-2X/def2-TZVP.

Figure S10. a) Activation strain analyses and b) energy decomposition analyses of the Diels-Alder reactions between the **CP** and **NMe**, $\mathbf{N(C_4H_8)}^+$, $\mathbf{N(C_4H_8O)}^+$, and $\mathbf{NMe_2}^+$ going from the reactants to the product, where the transition states are indicated with a dot and the energy values are projected onto the shorter newly forming $C_{\mathbf{CP}}$•••C_β bond between **CP** and the dienophile, computed at ZORA-M06-2X/TZ2P//M06-2X/def2-TZVP.

Figure S11. Activation barriers for the Diels-Alder reactions versus the degree of asynchronicity ($\Delta r^{TS}_{C\cdots C}$), computed at M06-2X/def2-TZVP.

Figure S12. a) Activation strain analyses and b) energy decomposition analyses of the asynchronous (black) and constraint synchronous (red) Diels-Alder reactions between the **CP** and **NMe** going from the reactants to the product, where the energy values are projected onto the shorter newly forming $C_{\mathbf{CP}}$•••C_β between **CP** and the **NMe**, computed at ZORA-M06-2X/TZ2P//M06-2X/def2-TZVP for $\mathbf{NMe_{asynch}}$ and ZORA-M06-2X/TZ2P for $\mathbf{NMe_{synch}}$.

Figure S13. a) Activation strain analyses and b) energy decomposition analyses of the asynchronous (black) and constraint synchronous (red) Diels-Alder reactions between the **CP** and $\mathbf{N(C_4H_8O)}^+$ going from the reactants to the product, where the energy values are projected onto the shorter newly forming $C_{\mathbf{CP}}$•••C_β between **CP** and the $\mathbf{N(C_4H_8O)}^+$, computed at ZORA-M06-2X/TZ2P//M06-2X/def2-TZVP for $\mathbf{N(C_4H_8O)}^+_{\mathbf{asynch}}$ and ZORA-M06-2X/TZ2P for $\mathbf{N(C_4H_8O)}^+_{\mathbf{synch}}$.

Figure S14. Occupied-occupied orbital overlap of a) $\text{HOMO}_{\mathbf{NMe2+}}$ with HOMO–$1_{\mathbf{CP}}$ and HOMO–$6_{\mathbf{CP}}$ and b) HOMO–$1_{\mathbf{NMe2+}}$ with HOMO–$1_{\mathbf{CP}}$ and HOMO–$6_{\mathbf{CP}}$ and c) the representation of the orbitals (isovalue = 0.03 au$^{-3/2}$) of the asynchronous (black) and constraint synchronous (red) Diels-Alder reactions between the **CP** and $\mathbf{NMe_2}^+$ going from the reactants to the product, where the orbital overlap values are projected onto the shorter newly forming $C_{\mathbf{CP}}$•••C_β bond between the **CP** and the $\mathbf{NMe_2}^+$, computed at ZORA-M06-2X/TZ2P//M06-2X/def2-TZVP for $\mathbf{NMe_2}^+_{\mathbf{asynch}}$ and ZORA-M06-2X/TZ2P for $\mathbf{NMe_2}^+_{\mathbf{synch}}$.

Figure S15. Key unoccupied orbitals (isovalue = 0.03 au$^{-3/2}$) computed at the equilibrium structures of **NMe**, $\mathbf{N(C_4H_8O)}^+$, and $\mathbf{NMe_2}^+$, where the MO-coefficients of the carbon and nitrogen $2p_z$ atomic orbitals, contributing to the LUMO, are shown in the schematic LUMOs.

Figure S16. a) The normal electron demand, $\langle \text{HOMO}_{\mathbf{CP}}|\text{LUMO}_{\mathbf{NMe2+}}\rangle$, and inverse electron demand, $\langle \text{HOMO}_{\mathbf{NMe2+}}|\text{LUMO}_{\mathbf{CP}}\rangle$, orbital overlap and b) the representation of the orbitals (isovalue = 0.03 au$^{-3/2}$) of the asynchronous (black) and constraint synchronous (red) Diels-Alder reactions between the **CP** and $\mathbf{NMe_2}^+$ going from the reactants to the product, where the orbital overlap values are projected onto the shorter newly forming $C_{\mathbf{CP}}$•••C_β bond between the **CP** and the $\mathbf{NMe_2}^+$ computed at ZORA-M06-2X/TZ2P//M06-2X/def2-TZVP for $\mathbf{NMe_2}^+_{\mathbf{asynch}}$ and ZORA-M06-2X/TZ2P for $\mathbf{NMe_2}^+_{\mathbf{synch}}$.

Table S2. Cartesian coordinates (in Å), energies (in kcal mol^{-1}), and number of imaginary frequencies of all stationary points, computed at M06-2X/def2-TZVP.

Computational Details

All stationary points and vibrational analyses were carried out at the M06-2X[1]/def2-TZVP[2] level using Gaussian 16.[3] The activation strain and energy decomposition analyses were carried out by using the PyFrag 2019[4] and ADF.2018.104[5] programs using the same functional in conjunction with the triple-ζ quality TZ2P basis set[6] on the geometries optimized at M06-2X/def2-TZVP. The zeroth-order regular approximation (ZORA) was used to account for scalar relativistic effects.[7] This level is referred to as ZORA-M06-2X/TZ2P//M06-2X/def2-TZVP. The Domain-Based Local Pair Natural Orbital Coupled-Cluster (DLPNO-CCSD(T))[8] calculations were performed using Orca 4.0.1[9] using the def2-QZVPP basis set on M06-2X/def2-TZVP geometries. Moreover, the NOCV (Natural Orbital for Chemical Valence) extension of the EDA method has also been used for further partitioning of the ΔE_{oi} term. The EDA-NOCV approach identifies the main molecular orbital interactions that dominate the total orbital interactions.[10]

---

[1] Y. Zhao, D. G. Truhlar, *Theor. Chem. Acc.* **2008**, *120*, 215.

[2] a) F. Weigend, R. Ahlrichs, *Phys. Chem. Chem. Phys.* **2005**, *7*, 3297; b) F. Weigend, *Phys. Chem. Chem. Phys.* **2006**, *8*, 1057.

[3] Gaussian 16, Revision B.01, M. J. Frisch, G. W. Trucks, H. B. Schlegel, G. E. Scuseria, M. A. Robb, J. R. Cheeseman, G. Scalmani, V. Barone, G. A. Petersson, H. Nakatsuji, X. Li, M. Caricato, A. V. Marenich, J. Bloino, B. G. Janesko, R. Gomperts, B. Mennucci, H. P. Hratchian, J. V. Ortiz, A. F. Izmaylov, J. L. Sonnenberg, D. Williams-Young, F. Ding, F. Lipparini, F. Egidi, J. Goings, B. Peng, A. Petrone, T. Henderson, D. Ranasinghe, V. G. Zakrzewski, J. Gao, N. Rega, G. Zheng, W. Liang, M. Hada, M. Ehara, K. Toyota, R. Fukuda, J. Hasegawa, M. Ishida, T. Nakajima, Y. Honda, O. Kitao, H. Nakai, T. Vreven, K. Throssell, J. A. Montgomery, Jr., J. E. Peralta, F. Ogliaro, M. J. Bearpark, J. J. Heyd, E. N. Brothers, K. N. Kudin, V. N. Staroverov, T. A. Keith, R. Kobayashi, J. Normand, K. Raghavachari, A. P. Rendell, J. C. Burant, S. S. Iyengar, J. Tomasi, M. Cossi, J. M. Millam, M. Klene, C. Adamo, R. Cammi, J. W. Ochterski, R. L. Martin, K. Morokuma, O. Farkas, J. B. Foresman, and D. J. Fox, Gaussian, Inc., Wallingford CT, 2016.

[4] X. Sun, T. M. Soini, J. Poater, T. A. Hamlin, F. M. Bickelhaupt, *J. Comp. Chem.*, **2019**, *40*, 2227.

[5] a) G. te Velde, F. M. Bickelhaupt, E. J. Baerends, C. Fonseca Guerra, S. J. A. van Gisbergen, J. G. Snijders, T. Ziegler, *J. Comput. Chem.* **2001**, *22*, 931; b) C. Fonseca Guerra, J. G. Snijders, G. te Velde, E. J. Baerends, *Theor. Chem. Acc.* **1998**, *99*, 391; c) ADF2018.104, SCM Theoretical Chemistry, Vrije Universiteit: Amsterdam (Netherlands). http:// www.scm.com.

[6] a) E. van Lenthe, E. J. Baerends, *J. Comput. Chem.* **2003**, *24*, 1142; b) M. Franchini, P. H. T. Philipsen, E. van Lenthe, L. Visscher, *J. Chem. Theory Comput.* **2014**, *10*, 1994.

[7] a) E. van Lenthe, E. J. Baerends, J. G. Snijders, *J. Chem. Phys.* **1993**, *99*, 4597; b) E. van Lenthe, E. J. Baerends, J. G. Snijders, *J. Chem. Phys.* **1994**, *101*, 9783.

[8] a) F. Neese, *WIREs Comput. Mol. Sci.* **2018**, *8*, e1327; b) C. Riplinger, B. Sandhoefer, A. Hansen, F. Neese, *J. Chem. Phys*. **2013**, *139*, 134101.

[9] a) C. Riplinger, B. Sandhoefer, A. Hansen, F. Neese, J. Chem. Phys. **2013**, *139*, 134101; b) F. Neese, *WIREs Comput. Mol. Sci.* **2018**, *8*, e1327.

[10] M. P. Mitoraj, A. Michalak, T. A. Ziegler, *J. Chem. Theory Comput.* **2009**, *5*, 962.

Table S1. Gibbs free reactant complexes (ΔG_{RC}), reaction barriers ($\Delta G^{\ddagger}$), reaction energies (ΔG_{rxn}) (in kcal mol^{-1}), computed for the Diels-Alder reactions between cyclopentadiene (**CP**) and various aldehyde, imine, and iminium dienophiles.[a]

X	cycloadduct	ΔG_{RC}	$\Delta G^{\ddagger}$	ΔG_{rxn}
O	*endo*	6.1	28.1	–7.2
	exo	6.9	28.7	–7.0
O–$AlCl_3$	*endo*	4.7	15.6	–8.3
	exo	6.0	16.3	–7.7
NMe	*endo*	6.4	31.5	–5.9
	exo	7.2	32.0	–5.7
MeN–$AlCl_3$	*endo*	6.1	24.0	–6.7
	exo	6.5	23.9	–7.9
$N(C_4H_8)^+$	*endo*	1.3	12.2	–10.4
	exo	2.6	12.6	–9.7
$N(C_4H_8O)^+$	*endo*	1.4	11.2	–11.0
	exo	2.4	12.6	–9.7
NMe_2^+	*endo*	0.8	10.7	–11.2
	exo	2.3	10.9	–10.4

[a] Computed at M06-2X/def2-TZVP

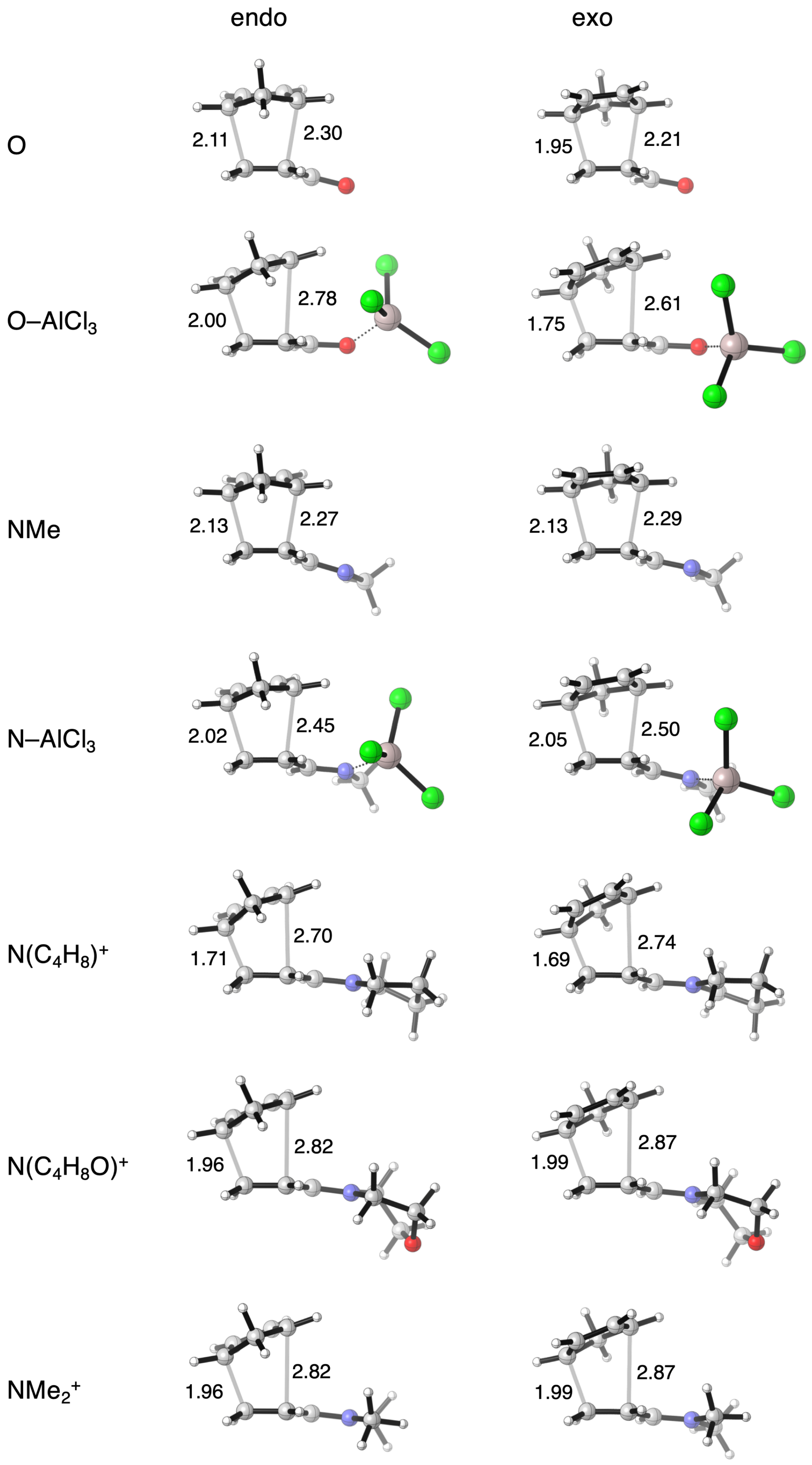

Figure S1. Transition state structures for the Diels–Alder reaction between cyclopentadiene and various dienophiles forming an endo (left) or exo (right) cycloadduct, computed at M06-2X/def2-TZVP.

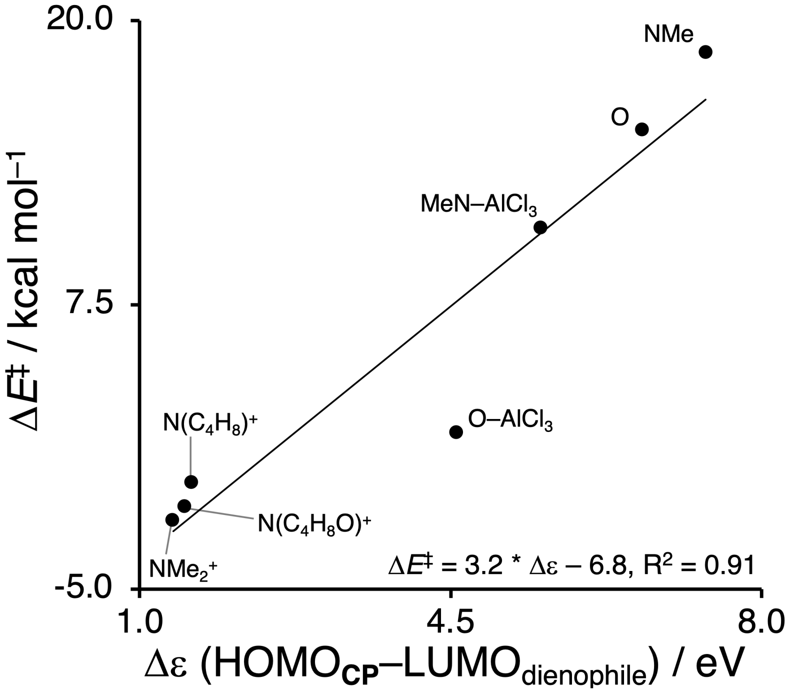

Figure S2: Activation barriers for the Diels-Alder reactions versus the reactants' $HOMO_{CP}$–$LUMO_{dienophile}$ gaps ($\Delta\varepsilon$), computed at M06-2X/def2-TZVP.

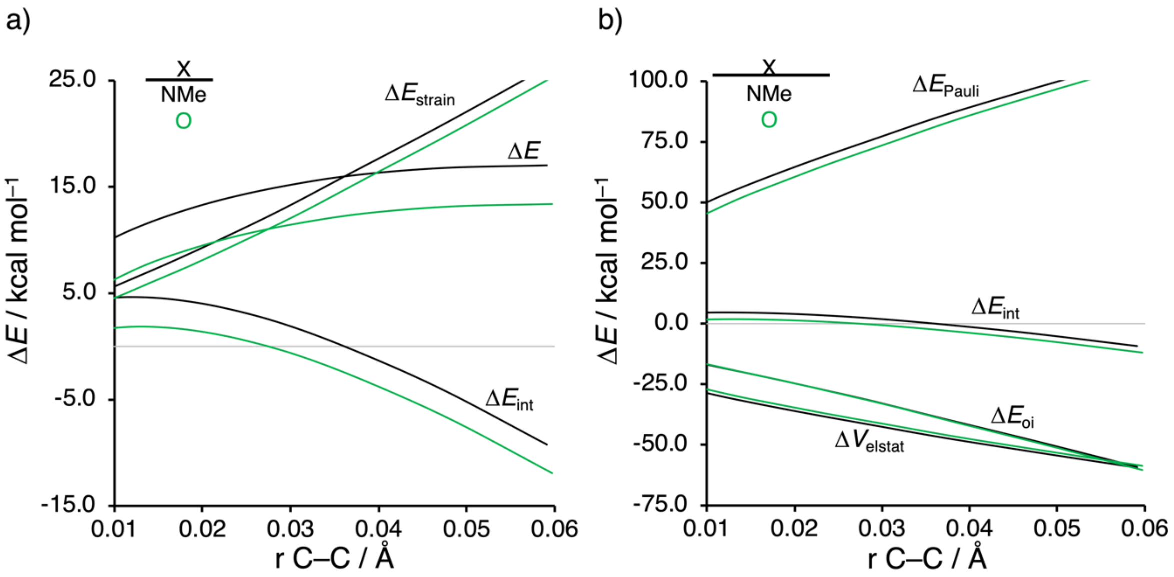

Figure S3 a) Activation strain analyses and b) energy decomposition analyses of the Diels-Alder reactions between the **CP** and **O** and **NMe** going from the reactants to the transition states, where the energy values are projected onto the stretch of the C_α•••C_β bond of the dienophile, computed at ZORA-M06-2X/TZ2P//M06-2X/def2-TZVP.

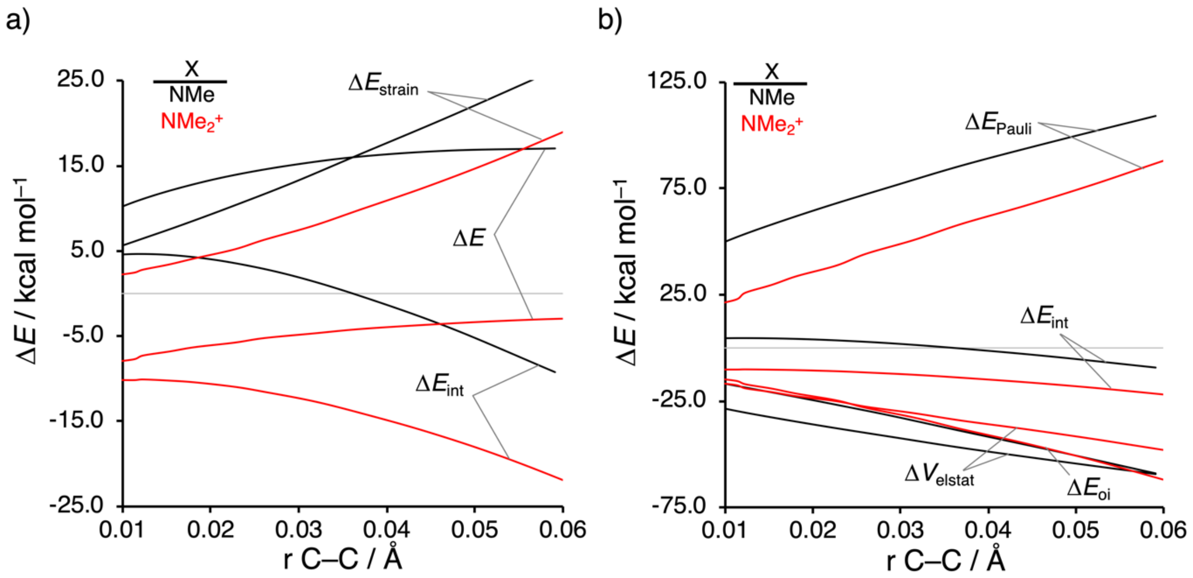

Figure S4. a) Activation strain analyses and b) energy decomposition analyses of the Diels-Alder reactions between the **CP** and **NMe** and **NMe_2^+** going from the reactants to the transition states, where the energy values are projected onto the stretch of the C_α•••C_β bond of the dienophile, computed at ZORA-M06-2X/TZ2P//M06-2X/def2-TZVP.

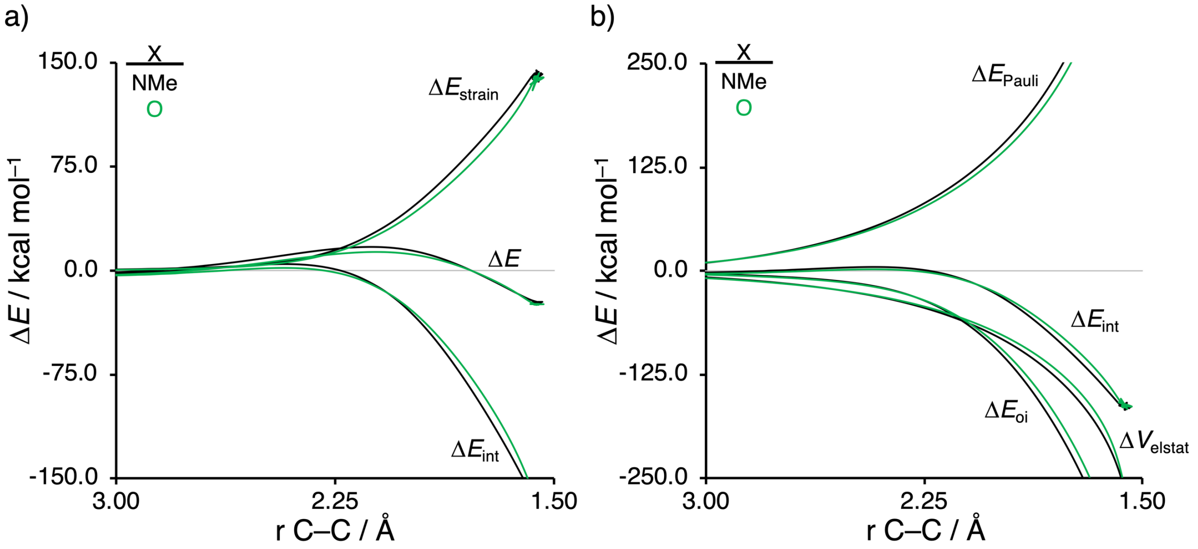

Figure S5. a) Activation strain analyses and b) energy decomposition analyses of the Diels-Alder reactions between the **CP** and **O** and **NMe** going from the reactants to the products, where the energy values are projected onto the shorter newly forming $C_{\mathbf{CP}}$•••C_{β} bond between **CP** and the dienophile, computed at ZORA-M06-2X/TZ2P//M06-2X/def2-TZVP.

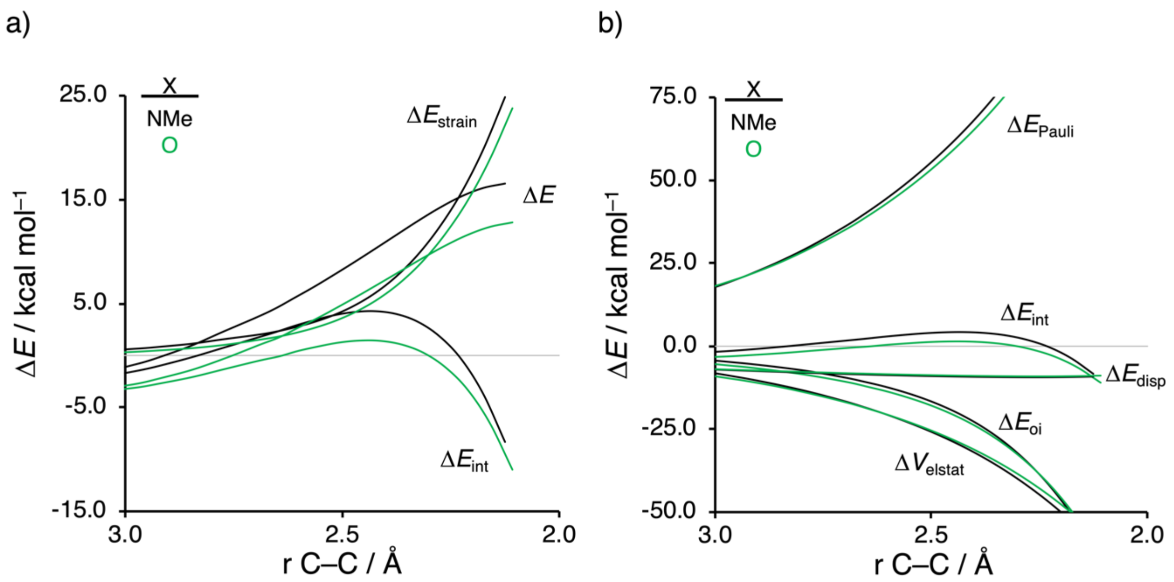

Figure S6. a) Activation strain analyses and b) energy decomposition analyses of the Diels-Alder reactions between the **CP** and **O** and **NMe** going from the reactants to the transition states, where the energy values are projected onto the shorter newly forming $C_{\mathbf{CP}}$••• C_{β} bond between the **CP** and the dienophile, computed at ZORA-BP86-D3(BJ)/TZ2P//M06-2X/def2-TZVP.

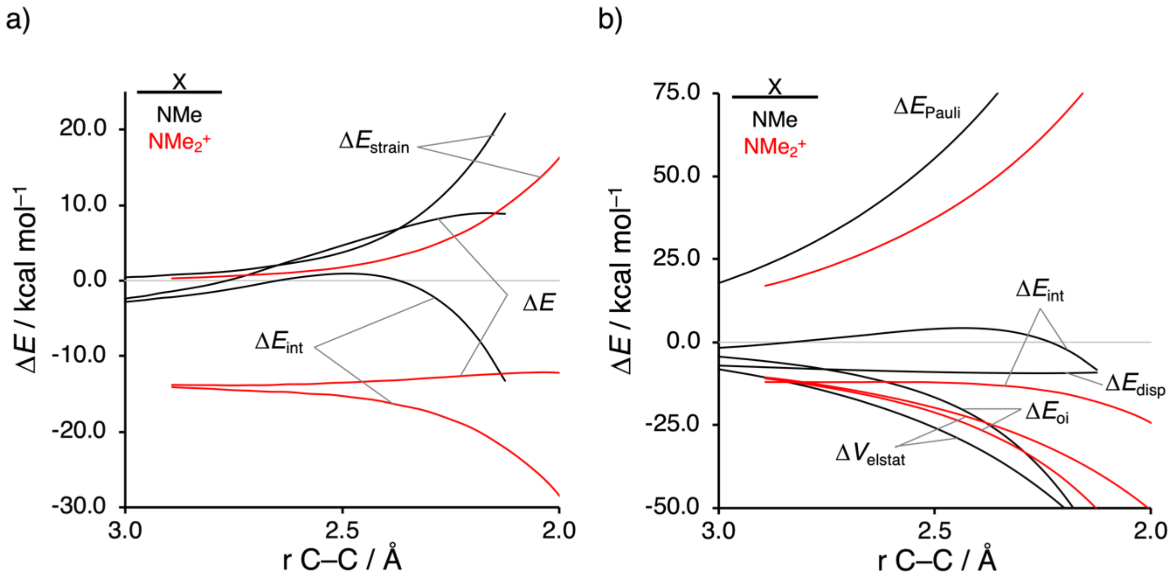

Figure S7. a) Activation strain analyses and b) energy decomposition analyses of the Diels-Alder reactions between the **CP** and **NMe** and $\mathbf{NMe_2}^{+}$ going from the reactants to the transition states, where the energy values are projected onto the shorter newly forming $C_{\mathbf{CP}}$••• C_{β} bond between **CP** and the dienophile, computed at ZORA-BP86-D3(BJ)/TZ2P//M06-2X/def2-TZVP.

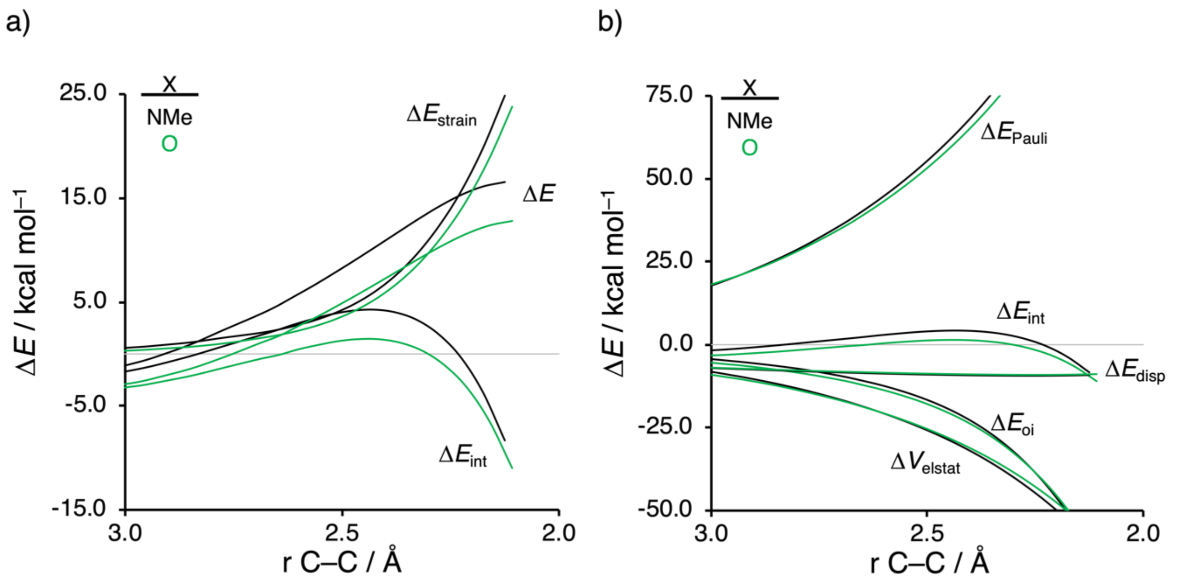

Figure S8. a) Activation strain analyses and b) energy decomposition analyses of the Diels-Alder reactions between the **CP** and **O** and **NMe** going from the reactants to the transition states, where the energy values are projected onto the shorter newly forming C_{CP}•••C_{β} bond between the **CP** and the dienophile, computed at ZORA-B3LYP-D3(BJ)/TZ2P//M06-2X/def2-TZVP.

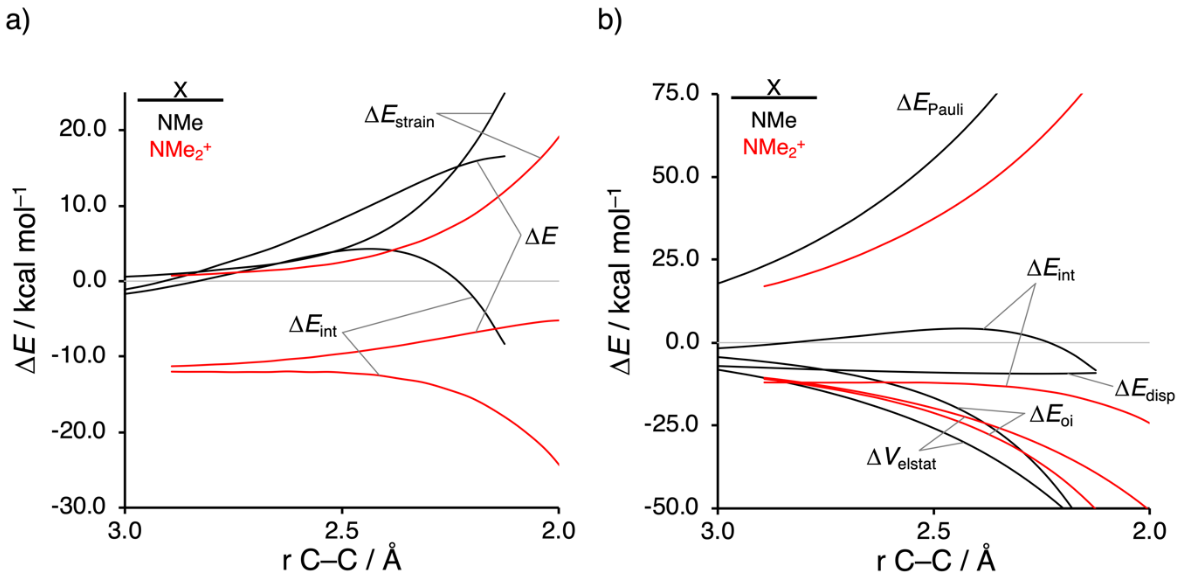

Figure S9. a) Activation strain analyses and b) energy decomposition analyses of the Diels-Alder reactions between the **CP** and **NMe** and **NMe_2^+** going from the reactants to the transition states, where the energy values are projected onto the shorter newly forming C_{CP}•••C_{β} bond between **CP** and the dienophile, computed at ZORA-BP86-D3(BJ)/TZ2P//M06-2X/def2-TZVP.

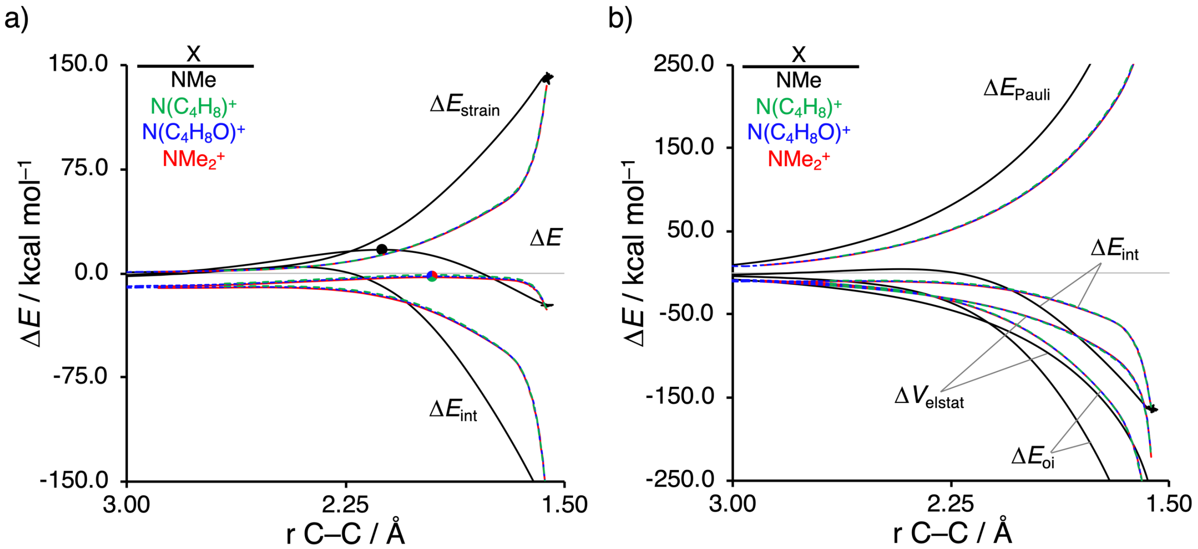

Figure S10. a) Activation strain analyses and b) energy decomposition analyses of the Diels-Alder reactions between the **CP** and **NMe**, **$N(C_4H_8)^+$**, **$N(C_4H_8O)^+$**, and **NMe_2^+** going from the reactants to the product, where the transition states are indicated with a dot and the energy values are projected onto the shorter newly forming $C_{CP}\cdots C_\beta$ bond between **CP** and the dienophile, computed at ZORA-M06-2X/TZ2P//M06-2X/def2-TZVP.

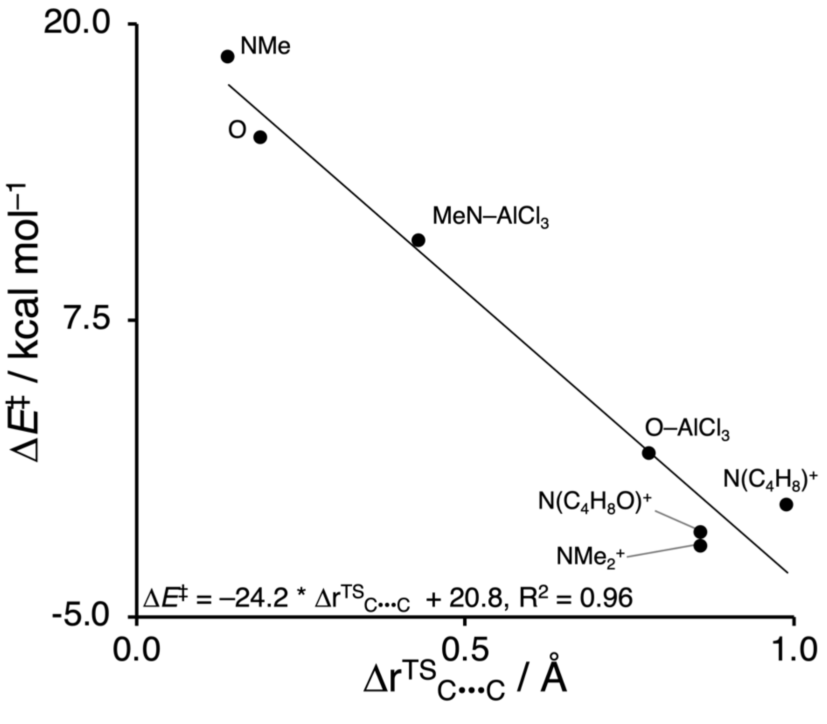

Figure S11. Activation barriers for the Diels-Alder reactions versus the degree of asynchronicity ($\Delta r^{TS}_{C\cdots C}$), computed at M06-2X/def2-TZVP.

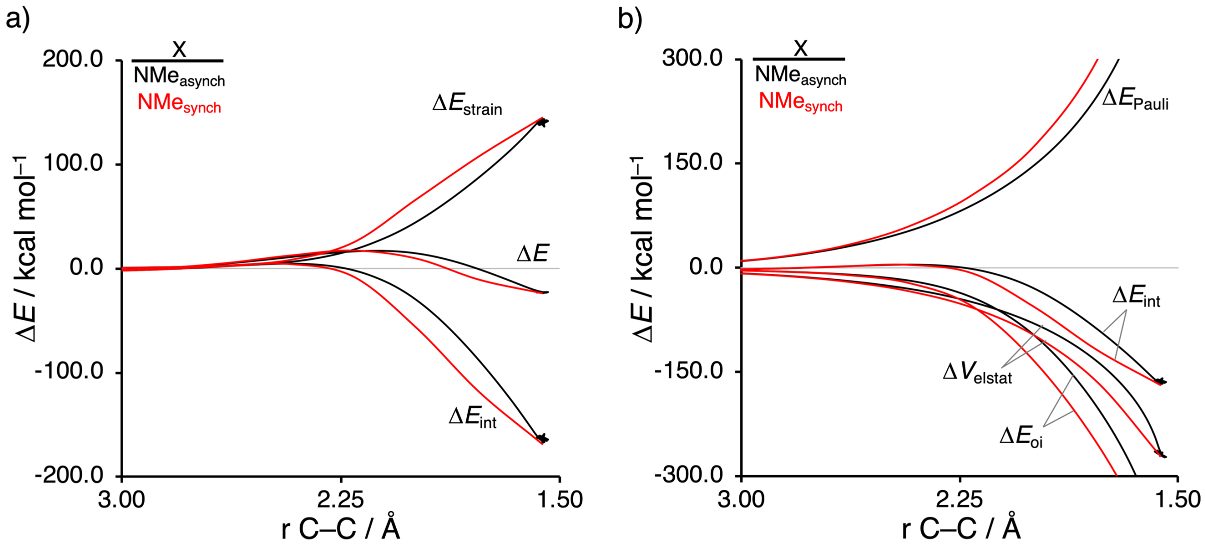

Figure S12. a) Activation strain analyses and b) energy decomposition analyses of the asynchronous (black) and constraint synchronous (red) Diels-Alder reactions between the **CP** and **NMe** going from the reactants to the product, where the energy values are projected onto the shorter newly forming C_{CP}•••C_{β} between **CP** and the **NMe**, computed at ZORA-M06-2X/TZ2P//M06-2X/def2-TZVP for **NMe$_{asynch}$** and ZORA-M06-2X/TZ2P for **NMe$_{synch}$**.

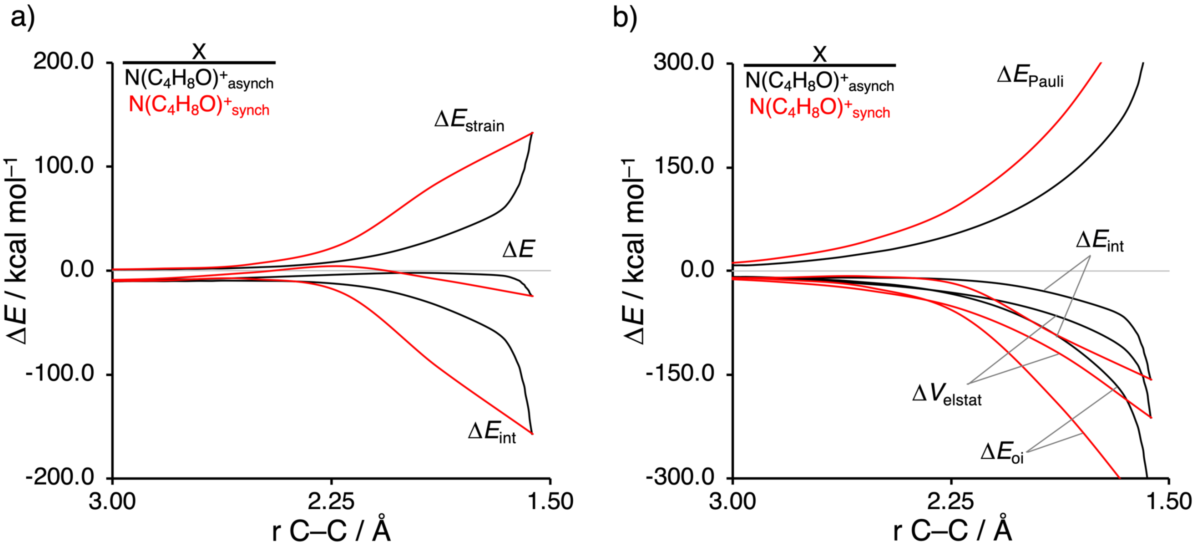

Figure S13. a) Activation strain analyses and b) energy decomposition analyses of the asynchronous (black) and constraint synchronous (red) Diels-Alder reactions between the **CP** and **$N(C_4H_8O)^+$** going from the reactants to the product, where the energy values are projected onto the shorter newly forming C_{CP}•••C_{β} between **CP** and the **$N(C_4H_8O)^+$**, computed at ZORA-M06-2X/TZ2P//M06-2X/def2-TZVP for **$N(C_4H_8O)^+_{asynch}$** and ZORA-M06-2X/TZ2P for **$N(C_4H_8O)^+_{synch}$**.

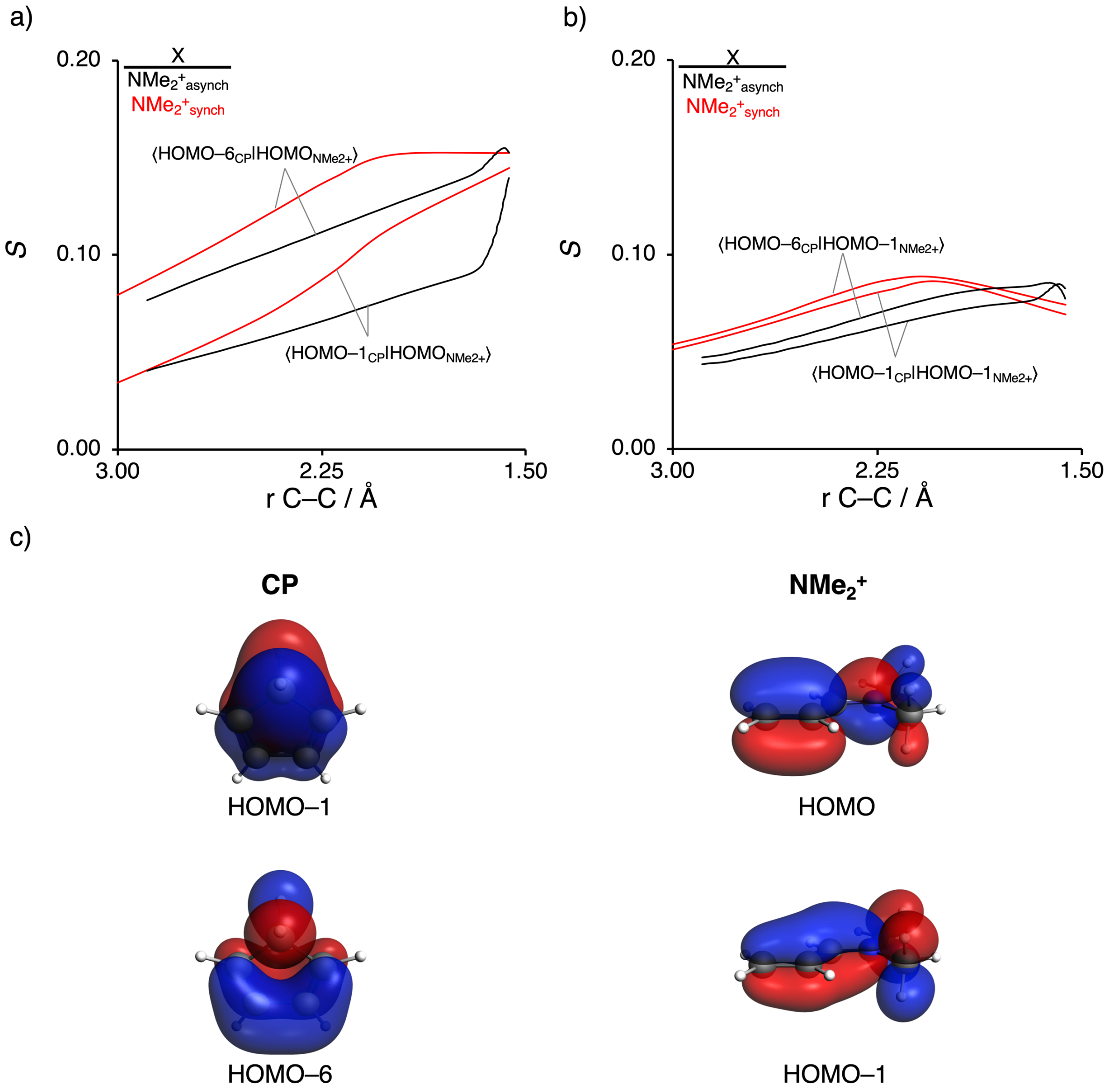

Figure S14. Occupied-occupied orbital overlap of a) $\text{HOMO}_{\textbf{NMe2+}}$ with $\text{HOMO–1}_{\textbf{CP}}$ and $\text{HOMO–6}_{\textbf{CP}}$ and b) $\text{HOMO–1}_{\textbf{NMe2+}}$ with $\text{HOMO–1}_{\textbf{CP}}$ and $\text{HOMO–6}_{\textbf{CP}}$ and c) the representation of the orbitals (isovalue = 0.03 au$^{-3/2}$) of the asynchronous (black) and constraint synchronous (red) Diels-Alder reactions between the **CP** and $\textbf{NMe}_2^+$ going from the reactants to the product, where the orbital overlap values are projected onto the shorter newly forming $C_{\textbf{CP}}$•••C_β bond between **CP** and the $\textbf{NMe}_2^+$, computed at ZORA-M06-2X/TZ2P//M06-2X/def2-TZVP for $\textbf{NMe}_2{}^+{}_{\textbf{asynch}}$ and ZORA-M06-2X/TZ2P for $\textbf{NMe}_2{}^+{}_{\textbf{synch}}$.

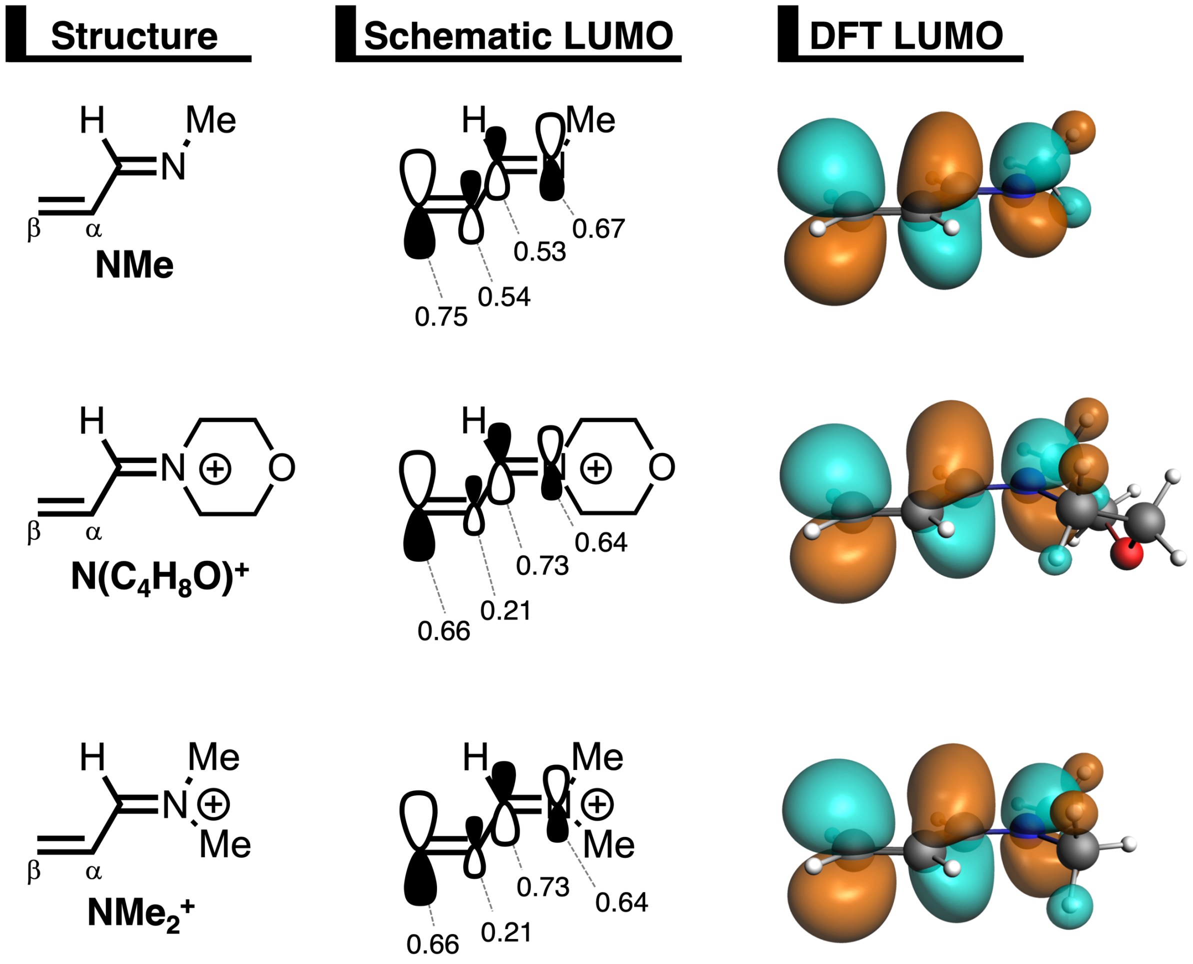

Figure S15. Key unoccupied orbitals (isovalue = 0.03 au$^{-3/2}$) computed at the equilibrium structures of **NMe**, **N(C$_4$H$_8$O)**$^+$, and **NMe$_2$**$^+$, where the MO-coefficients of the carbon and nitrogen 2p_z atomic orbitals, contributing to the LUMO, are shown in the schematic LUMOs.

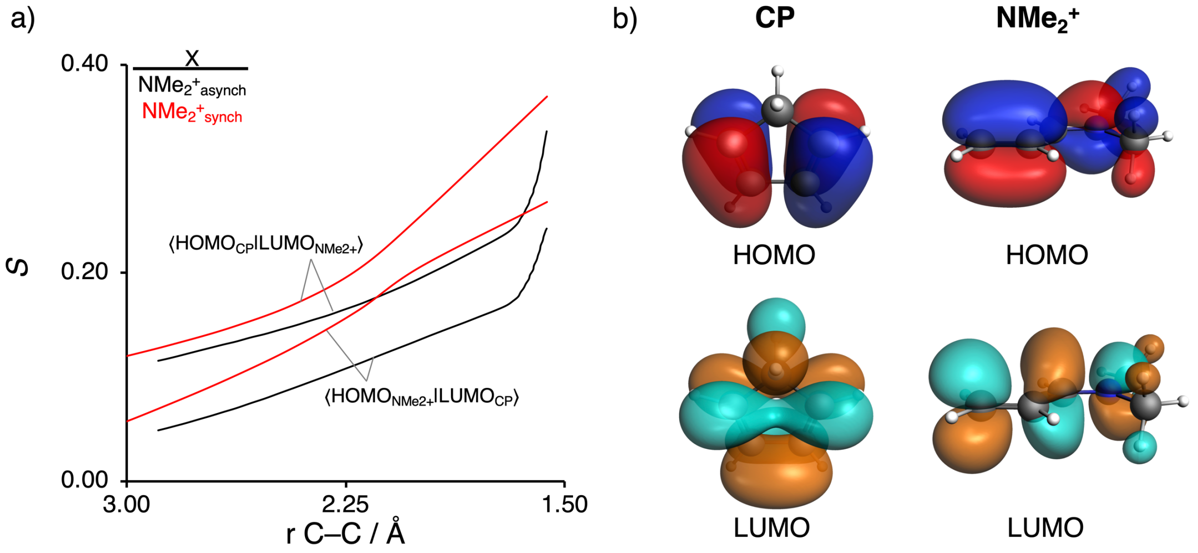

Figure S16. a) The normal electron demand, ⟨$HOMO_{\mathbf{CP}}$|$LUMO_{\mathbf{NMe2+}}$⟩, and inverse electron demand, ⟨$HOMO_{\mathbf{NMe2+}}$|$LUMO_{\mathbf{CP}}$⟩, orbital overlap and b) the representation of the orbitals (isovalue = 0.03 au$^{-3/2}$) of the asynchronous (black) and constraint synchronous (red) Diels-Alder reactions between the **CP** and $\mathbf{NMe_2}^+$ going from the reactants to the product, where the orbital overlap values are projected onto the shorter newly forming $C_{\mathbf{CP}}$•••C_β bond between **CP** and the $\mathbf{NMe_2}^+$, computed at ZORA-M06-2X/TZ2P//M06-2X/def2-TZVP for $\mathbf{NMe_2}^+_{\mathbf{asynch}}$ and ZORA-M06-2X/TZ2P for $\mathbf{NMe_2}^+_{\mathbf{synch}}$.

Table S2. Cartesian coordinates (in Å), energies (in Hartree), and number of imaginary frequencies of all stationary points, computed at M06-2X/def2-TZVP.

cyclopentadiene (CP)
E = −193.986966
H = −193.981893
G = −194.013547
N_{imag} = 0

C	-1.17369	-0.27961	0.00000
C	-0.73253	0.98523	-0.00000
C	0.73422	0.98396	-0.00000
C	1.17321	-0.28163	0.00000
C	-0.00104	-1.21228	0.00000
H	-2.20333	-0.60434	0.00000
H	-1.34593	1.87487	-0.00000
H	1.34916	1.87255	-0.00000
H	2.20228	-0.60814	0.00000
H	-0.00161	-1.86927	-0.87653
H	-0.00161	-1.86926	0.87653

Acrylaldehyde (O)
E = −191.839556
H = −191.834262
G = −191.865841
N_{imag} = 0

O	-1.78077	-0.12129	0.00010
C	-0.67513	0.34826	0.00002
H	-0.51946	1.44582	-0.00007
C	0.56370	-0.45298	0.00003
C	1.74833	0.14452	-0.00006
H	0.44701	-1.53022	0.00012
H	2.67644	-0.41114	-0.00005
H	1.82080	1.22707	-0.00015

Acrylaldehyde-$AlCl_3$ (O–$AlCl_3$)
E = −1815.123069
H = −1815.112414
G = −1815.161974
N_{imag} = 0

O	0.84920	-0.00168	0.67491
C	1.92095	-0.00035	0.07262
H	1.91943	0.00209	-1.02408
C	3.18795	-0.00188	0.76845
C	4.30971	-0.00031	0.05021
H	3.17002	-0.00429	1.85055
H	5.28615	-0.00133	0.51588
H	4.27744	0.00210	-1.03356
Al	-0.90813	0.00010	-0.02033
Cl	-0.51385	-0.00081	-2.10734
Cl	-1.68654	1.79693	0.73578
Cl	-1.69093	-1.79441	0.73676

***N*-methylprop-2-en-1-imine (NMe)**
E = −211.220010
H = −211.213211
G = −211.248556
N_{imag} = 0

N	1.06796	-0.49817	0.00010
C	0.01065	0.19161	-0.00014
H	0.03304	1.29123	-0.00059
C	-1.31298	-0.43090	-0.00013
C	-2.43293	0.28241	0.00014
H	-1.32521	-1.51467	-0.00032
H	-3.40783	-0.18572	0.00022
H	-2.40538	1.36637	0.00023
C	2.33316	0.19876	0.00007
H	2.90616	-0.10527	0.87755
H	2.90683	-0.10654	-0.87658
H	2.22934	1.29044	-0.00089

***N*-methylprop-2-en-1-imine−$AlCl_3$ (MeN−$AlCl_3$)**
E = −1834.517811
H = −1834.504903
G = −1834.557380
N_{imag} = 0

C	-2.51046	-0.11392	-0.00060
C	-3.84012	-0.07046	-0.00059
C	-1.76497	1.13133	-0.00109
N	-0.49278	1.23738	-0.00080
H	-2.36158	2.04541	-0.00155
H	-1.97416	-1.05315	0.00000
H	-4.43402	-0.97403	-0.00008
H	-4.37410	0.87309	-0.00120
Al	0.74847	-0.30168	0.00027
Cl	0.26248	-1.34222	-1.77808
Cl	0.26206	-1.33838	1.78079
Cl	2.66271	0.59361	-0.00104
C	0.09790	2.57632	-0.00102
H	0.73253	2.68417	-0.87924
H	-0.67947	3.33945	-0.00110
H	0.73261	2.68446	0.87710

1-allylidenepyrrolidin-1-ium ($N(C_4H_8)^+$)
E = −328.242359
H = −328.233543
G = −328.274845
N_{imag} = 0

C	-2.22797	0.41168	0.04838
C	-3.48759	-0.03025	0.05118
C	-1.15142	-0.53792	-0.05394
H	-1.39872	-1.59452	-0.11779
N	0.09441	-0.23865	-0.07184
H	-2.00083	1.46650	0.12573

H	-3.71812	-1.08675	-0.02441
H	-4.32362	0.65203	0.12822
C	1.18846	-1.23995	-0.15866
H	0.92229	-2.12067	0.42095
H	1.30253	-1.51493	-1.20891
C	0.64893	1.13733	0.02094
H	0.17391	1.77294	-0.72372
H	0.42261	1.51929	1.01787
C	2.14727	0.93919	-0.19068
H	2.72605	1.70334	0.32148
H	2.38520	0.98743	-1.25372
C	2.39927	-0.47020	0.34758
H	2.41631	-0.46976	1.43825
H	3.32968	-0.90356	-0.00994

4-allylidenemorpholin-4-ium ($N(C_4H_8O)^+$)

E = −403.440615

H = −403.431076

G = −403.4743.49

N_{imag} = 0

C	2.45758	0.40435	-0.10364
C	3.67412	-0.08828	-0.35073
H	4.51891	0.56335	-0.53048
C	1.37515	-0.51281	0.13311
N	0.15214	-0.19219	0.36291
H	1.60193	-1.57521	0.13156
H	2.28340	1.47044	-0.06657
H	3.85959	-1.15592	-0.38077
C	-0.88452	-1.19909	0.63196
H	-0.43657	-2.18950	0.61651
H	-1.25404	-1.00364	1.63936
C	-0.33537	1.20843	0.36993
H	-0.05558	1.67478	1.31689
H	0.14870	1.72882	-0.45344
C	-1.84720	1.19880	0.14512
H	-2.39581	0.97697	1.06637
H	-2.16299	2.17837	-0.20649
C	-2.00658	-1.06788	-0.41202
H	-1.78728	-1.67337	-1.28972
H	-2.94139	-1.41833	0.03556
O	-2.13036	0.26344	-0.86270

***N*-allylidene-*N*-methylmethanaminium (NMe_2^+)**

E = −250.854406

H = −250.846057

G = −250.885630

N_{imag} = 0

N	0.86527	-0.05792	-0.00004
C	-0.34544	-0.49102	0.00012
H	-0.47450	-1.56917	0.00024
C	-1.52328	0.33274	0.00019

C	-2.72231	-0.25618	-0.00015
H	-1.42822	1.40994	0.00046
H	-3.63451	0.32567	-0.00021
H	-2.82535	-1.33535	-0.00035
C	2.01088	-0.97308	-0.00001
H	2.61094	-0.78135	-0.88861
H	2.61081	-0.78146	0.88870
H	1.66181	-2.00127	-0.00011
C	1.18315	1.37966	-0.00007
H	0.77112	1.84710	0.89251
H	2.26240	1.49150	-0.00039
H	0.77065	1.84721	-0.89238

RC: endo_CP + O
E = −385.832276
H = −385.821190
G = −385.6869713
N_{imag} = 0

C	-1.66322	0.99063	0.35966
C	-0.89589	1.89756	-0.23172
H	-0.55727	2.79112	0.27522
C	-2.07952	-0.22252	-0.36635
O	-2.70132	-1.12445	0.13190
H	-1.77432	-0.25498	-1.42998
H	-1.99678	1.08180	1.38715
H	-0.57154	1.75871	-1.25748
C	0.86521	-1.33550	-0.19670
C	1.43366	-0.59731	-1.16103
C	2.13829	0.53360	-0.54889
C	1.99084	0.47436	0.78273
C	1.17247	-0.72786	1.13675
H	0.27411	-2.22981	-0.32841
H	1.39156	-0.79402	-2.22321
H	2.68856	1.28556	-1.09685
H	2.39823	1.16353	1.50754
H	0.26091	-0.46301	1.68443
H	1.72605	-1.42106	1.77965

RC: exo_CP + O
E = −385.831196
H = −385.820114
G = −385.868375
N_{imag} = 0

C	-2.18607	0.46057	-0.57952
C	-1.47565	-0.69771	-1.12633
C	-0.84891	-1.34108	-0.13090
C	-1.98766	0.51439	0.74583
C	-1.13160	-0.64180	1.16279
H	-2.77549	1.15299	-1.16390
H	-1.46295	-0.97510	-2.17075
H	-0.23157	-2.22285	-0.21798

H	-2.38924	1.24793	1.42928
H	-0.21681	-0.32064	1.67279
H	-1.65709	-1.29801	1.86574
C	1.48350	0.85471	-0.53903
C	0.92103	1.92703	0.00540
C	2.21307	-0.10584	0.30523
H	2.26877	0.17263	1.37841
O	2.71937	-1.12061	-0.09423
H	1.41864	0.62650	-1.59561
H	1.00106	2.11226	1.07193
H	0.36348	2.64754	-0.57691

TS: endo_CP + O
E = −385.802226
H = −385.793468
G = −385.834579
N_{imag} = 1,-481.1559i cm^{-1}

C	-0.92613	0.85204	0.31237
C	-0.00399	1.60845	-0.39495
H	0.40892	2.50164	0.05544
C	-1.92093	0.03935	-0.38639
O	-2.91578	-0.42738	0.11104
H	-1.68871	-0.13770	-1.45816
H	-1.13853	1.05902	1.35381
H	-0.06645	1.63548	-1.47507
C	0.39926	-0.95504	0.84559
C	0.66521	-1.42732	-0.43222
C	1.53078	-0.53572	-1.07048
C	1.79941	0.53234	-0.21316
C	1.44932	0.06189	1.17339
H	-0.20678	-1.46251	1.58261
H	0.18328	-2.27591	-0.89573
H	1.83335	-0.59161	-2.10658
H	2.55838	1.27823	-0.40527
H	1.16430	0.83190	1.88456
H	2.32091	-0.47551	1.57109

TS: exo_CP + O
E = −385.801339
H = −385.792592
G = −385.833701
N_{imag} = 1, -475.5260i cm^{-1}

C	-2.10929	-0.03921	-0.57449
C	-1.34643	-1.16998	-0.73778
C	-0.32665	-1.17058	0.23550
C	-1.55895	0.74187	0.48327
C	-0.78180	-0.24489	1.32468
H	-2.89568	0.29210	-1.23984
H	-1.41900	-1.87001	-1.55881
H	0.37338	-1.98045	0.38840
H	-2.11477	1.55526	0.93663

H	0.00529	0.18318	1.93946
H	-1.48407	-0.78409	1.97291
C	0.87872	0.45676	-0.64639
C	0.01409	1.54380	-0.34921
C	2.05776	0.20322	0.18120
H	2.07257	0.75600	1.14549
O	2.95773	-0.55188	-0.09465
H	0.93820	0.05830	-1.65090
H	0.32123	2.19319	0.46946
H	-0.42055	2.09338	-1.17725

P: endo_CP + O
E = −385.858868
H = −385.850756
G = −385.890820
N_{imag} = 0

C	-0.73708	0.50531	0.30646
C	0.17140	1.22734	-0.72629
H	0.15541	2.30395	-0.55984
C	-1.90649	-0.19726	-0.31698
O	-3.04420	-0.07610	0.04552
H	-1.65480	-0.86639	-1.16545
H	-1.12847	1.19844	1.05063
H	-0.13826	1.04434	-1.75622
C	0.26834	-0.48295	1.00229
C	0.73548	-1.47153	-0.04832
C	1.51998	-0.81222	-0.89842
C	1.58247	0.62926	-0.43830
C	1.49967	0.43542	1.08438
H	-0.11241	-0.90179	1.92974
H	0.39683	-2.49471	-0.13208
H	1.94622	-1.18723	-1.81854
H	2.40622	1.22417	-0.82337
H	1.30926	1.36191	1.62885
H	2.37104	-0.07409	1.49327

P: exo_CP + O
E = −385.858525
H = −385.850374
G = −385.890586
N_{imag} = 0

C	-2.10846	-0.16948	-0.59346
C	-1.41493	-1.28418	-0.37168
C	-0.23297	-0.91898	0.50204
C	-1.40238	0.95937	0.12790
C	-0.85123	0.20658	1.34908
H	-2.95412	-0.04768	-1.25560
H	-1.57071	-2.25567	-0.81900
H	0.25585	-1.73771	1.02433
H	-1.98835	1.85495	0.31606
H	-0.12234	0.78620	1.91792

H	-1.63480	-0.15883	2.01079
C	0.71981	-0.10107	-0.44787
C	-0.07839	1.21539	-0.65233
H	-0.24542	1.42953	-1.70578
C	2.05301	0.13311	0.20234
O	3.09685	-0.28379	-0.21648
H	2.02824	0.72755	1.14052
H	0.89937	-0.63915	-1.37657
H	0.45072	2.06672	-0.21697

RC: endo_CP + O-AlCl$_3$

E = −2009.121596
H = −2009.104607
G = −2009.167984
N_{imag} = 0

C	-1.11061	-1.81564	-0.12502
C	-2.32556	-2.33209	-0.32804
C	-0.51278	-1.06384	-1.19786
O	0.58840	-0.50576	-1.17440
H	-1.06685	-1.02310	-2.14134
Al	1.85573	0.10646	0.03044
Cl	0.94194	-0.03688	1.95090
Cl	2.08042	2.11008	-0.58440
Cl	3.47676	-1.19951	-0.25731
H	-0.56834	-1.91668	0.80526
H	-2.83839	-2.90324	0.43442
H	-2.83791	-2.20352	-1.27387
C	-1.93283	1.29589	0.03447
C	-2.69725	1.13978	-1.06245
C	-3.92350	0.43423	-0.69582
C	-3.89926	0.16397	0.62153
C	-2.64318	0.71724	1.21586
H	-0.97387	1.79359	0.09140
H	-2.46107	1.49526	-2.05609
H	-4.72112	0.18599	-1.38224
H	-4.67386	-0.32927	1.19043
H	-2.04006	-0.02566	1.74692
H	-2.86533	1.49859	1.95239

RC: exo_CP + O-AlCl$_3$

E = −2009.119316
H = −2009.102182
G = −2009.166023
N_{imag} = 0

C	-3.79200	-0.25563	-1.01906
C	-2.62318	-1.12371	-0.90846
C	-2.38389	-1.37824	0.38836
C	-4.26150	0.01789	0.21060
C	-3.43169	-0.70780	1.22426
H	-4.20788	0.10008	-1.95124
H	-2.03944	-1.48791	-1.74069

H	-1.58823	-1.99548	0.78065
H	-5.12704	0.61730	0.45258
H	-3.02065	-0.04599	1.99425
H	-4.03443	-1.44774	1.76371
C	-0.98186	1.50037	0.02734
C	-2.09653	2.22006	0.18861
C	-0.39237	0.90675	1.19583
O	0.66746	0.27479	1.24370
H	-0.92213	1.04431	2.14526
H	-0.50936	1.34508	-0.93418
H	-2.58612	2.70725	-0.64292
H	-2.53871	2.35772	1.16901
Al	1.96079	-0.12468	-0.04552
Cl	2.42675	1.79074	-0.81828
Cl	0.89620	-1.34321	-1.41591
Cl	3.47247	-1.08578	1.04365

TS: endo_CP + O–AlCl$_3$
E = −2009.107060
H = −2009.091645
G = −2009.150687
$\boldsymbol{N_{imag}}$ = 1,−349.2497i cm^{-1}

C	-1.34607	-1.50299	-0.46595
C	-2.71429	-1.66958	-0.71802
C	-0.54334	-0.82436	-1.37535
O	0.70494	-0.62871	-1.28588
H	-1.01761	-0.45413	-2.29169
Al	1.82217	0.00033	-0.02530
Cl	1.11502	-0.72495	1.86670
Cl	1.49157	2.11058	-0.13163
Cl	3.75716	-0.64368	-0.53085
H	-0.88757	-1.88617	0.43479
H	-3.23472	-2.45519	-0.18422
H	-3.06948	-1.52907	-1.73090
C	-1.77344	0.99962	0.67601
C	-2.33852	1.49053	-0.47424
C	-3.54577	0.80593	-0.70556
C	-3.70211	-0.21769	0.23242
C	-2.73103	0.08378	1.34781
H	-0.82681	1.30641	1.09917
H	-1.89691	2.24257	-1.11134
H	-4.20827	0.97674	-1.54372
H	-4.65481	-0.69856	0.40707
H	-2.27659	-0.75837	1.86262
H	-3.27115	0.67659	2.09961

TS: exo_CP + O-AlCl$_3$
E = −2009.106215
H = −2009.090900
G = −2009.149618
N_{imag} = 1,-345.2904i cm^{-1}

C	-3.57782	0.28055	1.05414
C	-2.56437	1.21083	0.94523
C	-2.18260	1.28184	-0.39126
C	-3.75823	-0.40080	-0.20192
C	-3.19569	0.58503	-1.22100
H	-4.06090	-0.01182	1.97736
H	-2.07028	1.72478	1.75562
H	-1.36766	1.88298	-0.77238
H	-4.71334	-0.88758	-0.39513
H	-2.82570	0.15835	-2.15090
H	-3.99047	1.30957	-1.46060
C	-1.22247	-1.13307	-0.11403
C	-2.55791	-1.67500	-0.20471
C	-0.46120	-0.89864	-1.23941
O	0.73199	-0.45261	-1.29458
H	-0.91130	-1.11200	-2.21562
H	-0.76805	-0.96165	0.85426
H	-2.84531	-2.33318	0.61939
H	-2.77412	-2.16146	-1.16189
Al	1.89549	0.03123	-0.00189
Cl	2.05950	-1.65975	1.27746
Cl	0.92095	1.66550	0.99495
Cl	3.66082	0.58696	-1.00442

P: endo_CP + O-AlCl$_3$
E = −2009.146043
H = −2009.131562
G = −2009.188779
N_{imag} = 0

C	-1.30820	0.06231	0.05338
C	-2.06966	-1.30856	0.02523
C	-0.53231	0.22225	-1.18204
O	0.69282	0.21989	-1.27831
H	-1.09485	0.32780	-2.11808
C	-2.48380	1.10071	0.19965
C	-3.34793	1.00834	-1.04125
C	-3.98031	-0.16314	-1.00232
C	-3.54665	-0.87682	0.26050
C	-3.35017	0.31971	1.20383
Al	2.02917	0.00047	0.03000
Cl	1.46999	-1.85651	0.89025
Cl	3.81358	0.00883	-1.06524
Cl	1.69150	1.66741	1.29171
H	-0.65380	0.11937	0.91987
H	-1.68126	-1.93813	0.82379
H	-1.94839	-1.85073	-0.91311

H	-2.13797	2.08809	0.49172
H	-3.36676	1.73513	-1.84145
H	-4.61340	-0.58939	-1.76794
H	-4.18548	-1.68626	0.60237
H	-2.82204	0.06643	2.12398
H	-4.28005	0.83835	1.42924

P: exo_CP + O–AlCl$_3$

E = −2009.145143

H = −2009.130628

G = −2009.187849

N_{imag} = 0

C	-3.75723	0.23510	1.04451
C	-2.78219	1.13546	0.93238
C	-2.11869	0.91784	-0.40710
C	-3.75484	-0.60843	-0.21192
C	-3.30802	0.43380	-1.24786
C	-1.32163	-0.45529	-0.18485
C	-2.47251	-1.49304	-0.14388
C	-0.41785	-0.62510	-1.32715
O	0.79896	-0.45108	-1.31807
H	-0.85868	-0.88217	-2.29931
Al	1.97750	0.02947	0.07168
Cl	1.11774	1.84534	0.75743
Cl	1.75773	-1.58413	1.42122
Cl	3.82975	0.20255	-0.88959
H	-4.37493	0.04893	1.91144
H	-2.41717	1.82274	1.68147
H	-1.50017	1.72816	-0.78127
H	-4.66174	-1.16783	-0.42275
H	-3.03279	-0.00298	-2.21012
H	-4.04226	1.22279	-1.39835
H	-0.74991	-0.40477	0.73751
H	-2.41602	-2.09827	0.75786
H	-2.43659	-2.16694	-1.00274

RC: endo_CP + NMe

E = −405.211976

H = −405.199169

G = −405.251950

N_{imag} = 0

C	-0.89978	1.68079	0.28492
C	0.08361	2.24887	-0.40297
H	0.67318	3.05897	0.00407
C	-1.68017	0.56684	-0.25164
N	-2.53470	-0.04495	0.44989
H	-1.46839	0.28344	-1.29210
H	-1.15532	2.00042	1.28913
H	0.33744	1.90301	-1.39872
C	0.88032	-1.38223	-0.21265
C	1.71269	-0.88181	-1.13646

C	2.68944	-0.00627	-0.47925
C	2.43988	0.01857	0.83727
C	1.26385	-0.85853	1.13623
H	0.05507	-2.05813	-0.38386
H	1.68473	-1.08444	-2.19801
H	3.47856	0.52730	-0.98991
H	2.98584	0.57235	1.58651
H	0.44227	-0.30256	1.60232
H	1.52337	-1.66696	1.82854
C	-3.23932	-1.14930	-0.15916
H	-2.94442	-1.33262	-1.19976
H	-3.05994	-2.05295	0.42677
H	-4.31252	-0.95477	-0.12183

RC: exo_CP + NMe
E = -405.210881
H = -405.198094
G = -405.250595
N_{imag} = 0

C	-2.84390	0.08594	-0.28745
C	-1.97245	-0.68870	-1.17545
C	-1.02872	-1.29826	-0.44474
C	-2.42412	-0.05848	0.97750
C	-1.23509	-0.96964	1.00139
H	-3.68751	0.67678	-0.61537
H	-2.08014	-0.74229	-2.24948
H	-0.22676	-1.92033	-0.81371
H	-2.86497	0.38443	1.85828
H	-0.35551	-0.48953	1.44296
H	-1.42910	-1.86957	1.59593
C	0.76327	1.38242	-0.51255
C	-0.00028	2.23021	0.16750
C	1.82500	0.61002	0.13051
H	1.98928	0.80953	1.20087
N	2.51078	-0.24412	-0.49834
H	0.62133	1.20678	-1.57183
H	0.14705	2.38655	1.23092
H	-0.79805	2.78611	-0.30504
C	3.53069	-0.96319	0.22911
H	3.59648	-0.67618	1.28569
H	3.32897	-2.03379	0.16216
H	4.49708	-0.79149	-0.24792

TS: endo_CP + NMe
E = -405.177350
H = -405.166891
G = -405.211984
N_{imag} = 1,-502.1991i cm^{-1}

C	0.15029	-1.10590	-0.35922
C	-1.00161	-1.26199	-1.15247
H	-1.48424	-2.23157	-1.13001

C	1.10198	-0.13910	-0.66952
N	2.27222	0.01783	-0.09383
H	0.88971	0.54904	-1.48289
H	0.32905	-1.78893	0.45883
H	-1.00771	-0.81259	-2.13687
C	-1.23489	0.73064	1.27782
C	-1.43158	1.58240	0.22337
C	-2.27562	0.94954	-0.71032
C	-2.53014	-0.36484	-0.30749
C	-2.13962	-0.44166	1.14769
H	-0.62332	0.93001	2.14707
H	-0.99637	2.56442	0.11137
H	-2.61303	1.38428	-1.64180
H	-3.35354	-0.93706	-0.71264
H	-1.74540	-1.38928	1.50433
H	-3.04262	-0.22355	1.73591
C	3.19830	1.06723	-0.49900
H	2.77651	1.62817	-1.32868
H	3.38275	1.74265	0.33726
H	4.14397	0.62364	-0.81043
C	2.67775	-0.84582	1.00650
H	3.67418	-0.56128	1.33084
H	1.98509	-0.73756	1.84421
H	2.69029	-1.88814	0.68624

TS: exo_CP + NMe

E = −405.176582

H = −405.166160

G = −405.211145

N_{imag} = 1,-499.2342i cm^{-1}

C	2.75719	0.38656	-0.54961
C	1.98501	1.49602	-0.13589
C	1.23067	1.12218	0.93944
C	2.41183	-0.73845	0.19794
C	1.64104	-0.23474	1.38978
H	3.45907	0.38961	-1.37288
H	1.97772	2.46479	-0.61258
H	0.52736	1.74913	1.47003
H	3.03862	-1.61901	0.24126
H	0.84304	-0.87058	1.76510
H	2.36232	-0.10788	2.20937
C	-0.15890	-0.56385	-0.92646
C	0.92522	-1.45453	-0.91912
C	-1.22455	-0.73509	-0.04931
H	-1.18586	-1.55545	0.66181
N	-2.32490	-0.01715	-0.01275
H	-0.19976	0.21877	-1.67014
H	0.82707	-2.36074	-0.33080
H	1.47948	-1.59504	-1.83633
C	-3.42060	-0.32674	0.89680
H	-3.16136	-1.19030	1.50342

H	-3.61468	0.52707	1.54648
H	-4.32238	-0.54880	0.32569
C	-2.49999	1.11579	-0.91101
H	-3.42237	1.62911	-0.65612
H	-1.66233	1.80776	-0.80301
H	-2.55314	0.77857	-1.94737

P: endo_CP + NMe

E = −405.237665

H = −405.227966

G = −405.271573

N_{imag} = 0

C	0.16922	-0.65098	0.30383
C	-0.72577	-1.13404	-0.87336
H	-0.82820	-2.21900	-0.85708
C	1.44998	-0.01757	-0.13050
N	2.57079	-0.39066	0.30624
H	1.36119	0.80234	-0.86035
H	0.42179	-1.48021	0.96411
H	-0.32152	-0.85152	-1.84595
C	-0.79105	0.32542	1.07028
C	-1.07932	1.49178	0.14585
C	-1.85390	1.04078	-0.83887
C	-2.09038	-0.43566	-0.59436
C	-2.10747	-0.45935	0.94348
H	-0.44417	0.57275	2.07014
H	-0.63700	2.47470	0.22961
H	-2.16749	1.57951	-1.72244
H	-2.93696	-0.88043	-1.11082
H	-2.04788	-1.46671	1.35953
H	-2.95741	0.07592	1.36466
C	3.75054	0.29615	-0.17300
H	3.52688	1.09480	-0.89079
H	4.28559	0.72103	0.67764
H	4.41858	-0.42779	-0.64199

P: exo_CP + NMe

E = −405.237127

H = −405.227371

G = −405.271194

N_{imag} = 0

C	-2.64451	-0.17175	-0.51648
C	-1.94056	-1.28581	-0.32805
C	-0.71711	-0.91990	0.48656
C	-1.90474	0.95755	0.17152
C	-1.29587	0.20267	1.36462
H	-3.51931	-0.04924	-1.13975
H	-2.11780	-2.25649	-0.76951
H	-0.20236	-1.73825	0.98396
H	-2.48282	1.85184	0.38947
H	-0.53876	0.78156	1.89664

H	-2.04691	-0.16120	2.06419
C	0.19397	-0.10049	-0.49738
C	-0.62089	1.21347	-0.67128
C	1.55771	0.13362	0.07077
H	1.60584	0.70121	1.01446
N	2.60375	-0.29658	-0.48197
H	0.32014	-0.63583	-1.43629
H	-0.07227	2.06695	-0.26681
H	-0.84202	1.42342	-1.71597
C	3.87326	-0.02876	0.15782
H	3.77840	0.54101	1.09041
H	4.37266	-0.97570	0.36809
H	4.51147	0.52318	-0.53372

RC: endo_CP + MeN-AlCl$_3$

E = −2028.51913

H = −2028.494129

G = −2028.561212

N_{imag} = 0

C	-1.17800	1.60471	-0.89595
C	-2.34344	2.23401	-1.03235
C	-0.47352	1.72255	0.36338
N	0.62220	1.15316	0.69383
H	-0.94319	2.38155	1.09660
Al	1.68988	-0.23849	-0.20098
Cl	1.04130	-0.65803	-2.17447
Cl	1.38413	-1.87949	1.11498
Cl	3.63587	0.59132	-0.12372
H	-0.74920	1.00774	-1.68719
H	-2.91390	2.17686	-1.94978
H	-2.76634	2.82228	-0.22642
C	-2.05138	-1.37666	-0.02586
C	-2.19955	-0.85481	1.20241
C	-3.47918	-0.14529	1.27221
C	-4.10110	-0.23807	0.08617
C	-3.26024	-1.05442	-0.84695
H	-1.21537	-1.96304	-0.37852
H	-1.49276	-0.95284	2.01544
H	-3.85777	0.35939	2.15039
H	-5.06867	0.16949	-0.16797
H	-3.00759	-0.52586	-1.77202
H	-3.78239	-1.96828	-1.15324
C	1.16797	1.45108	2.02517
H	2.14479	1.91739	1.90750
H	0.49937	2.11262	2.57404
H	1.29755	0.51771	2.57252

RC: exo_CP + MeN-AlCl$_3$
E = −2028.511890
H = −2028.493066
G = −2028.560632
***N*$_{imag}$** = 0

C	4.03690	-0.10274	1.05261
C	2.85644	-0.96232	1.14811
C	2.51700	-1.38132	-0.07949
C	4.40810	0.00083	-0.23295
C	3.48765	-0.82692	-1.07724
H	4.52485	0.36708	1.89501
H	2.33737	-1.20390	2.06399
H	1.68540	-2.02606	-0.32461
H	5.24923	0.55690	-0.62020
H	2.99882	-0.23620	-1.86059
H	4.03102	-1.62450	-1.59631
C	0.97954	1.48498	-0.12893
C	2.07234	2.22488	-0.30887
C	0.36607	0.85113	-1.27605
N	-0.72664	0.18623	-1.27728
H	0.89524	0.97039	-2.22375
H	0.54250	1.34330	0.85058
H	2.56223	2.72141	0.51660
H	2.50817	2.35954	-1.29264
Al	-1.87417	-0.07092	0.30386
Cl	-2.29557	1.89425	0.96861
Cl	-0.69398	-1.20856	1.64806
Cl	-3.55082	-1.11596	-0.45300
C	-1.18436	-0.39635	-2.53987
H	-2.16241	0.01092	-2.79073
H	-0.47345	-0.18692	-3.33853
H	-1.30004	-1.47209	-2.41446

TS: endo_CP + MeN-AlCl$_3$
E = −2028.487472
H = −2028.470657
G = −2028.532615
***N*$_{imag}$** = 1,−462.6389i cm^{-1}

C	-1.39938	0.04278	-1.28876
C	-2.70038	0.23599	-1.75735
C	-0.60012	1.16936	-0.94503
N	0.64101	1.18592	-0.58405
H	-1.10563	2.13653	-1.00565
Al	1.76554	-0.26653	0.03395
Cl	1.11983	-2.17683	-0.63813
Cl	1.50888	-0.06190	2.14564
Cl	3.70361	0.24683	-0.64626
H	-0.92114	-0.91995	-1.37261
H	-3.12434	-0.51695	-2.40918
H	-3.03117	1.24274	-1.97944

C	-2.01071	-0.51982	1.01925
C	-2.55701	0.71099	1.33673
C	-3.74739	0.87308	0.62169
C	-3.93221	-0.22802	-0.22403
C	-3.05484	-1.32634	0.32410
H	-1.09275	-0.92509	1.42341
H	-2.08706	1.45302	1.96630
H	-4.36307	1.76183	0.62250
H	-4.87021	-0.42978	-0.72359
H	-2.68970	-2.06463	-0.38421
H	-3.63119	-1.85003	1.09919
C	1.26032	2.49082	-0.32828
H	2.06391	2.65658	-1.04428
H	0.52146	3.28787	-0.40991
H	1.69264	2.50055	0.67335

TS: exo_CP + MeN-AlCl$_3$
E = −2028.488194
H = −2028.471405
G = −2028.532868
N_{imag} = 1,-440.7440i cm^{-1}

C	3.79437	-1.09415	0.14012
C	2.74560	-1.35938	-0.75089
C	2.29548	-0.16592	-1.27282
C	3.96771	0.28771	0.25391
C	3.29740	0.89294	-0.95205
H	4.30253	-1.83326	0.74345
H	2.28692	-2.32162	-0.92208
H	1.48860	-0.06234	-1.98386
H	4.82676	0.73885	0.73205
H	2.91144	1.90202	-0.83594
H	4.03751	0.90678	-1.76317
C	1.26223	0.42883	0.91863
C	2.45948	0.75714	1.55296
C	0.46856	1.44369	0.31950
N	-0.75347	1.33376	-0.09298
H	0.94039	2.42383	0.21670
H	0.82569	-0.54911	1.06217
H	2.79199	0.14953	2.38160
H	2.72635	1.80528	1.63696
Al	-1.80063	-0.29237	0.05196
Cl	-1.83202	-0.79093	2.11386
Cl	-0.77256	-1.73992	-1.13259
Cl	-3.69711	0.21833	-0.74402
C	-1.39454	2.51023	-0.68089
H	-2.27695	2.77759	-0.10079
H	-0.70168	3.35174	-0.70278
H	-1.72604	2.28129	-1.69311

P: endo_CP + MeN-AlCl$_3$
E = −2028.537703
H = −2028.521758
G = −2028.581607
N_{imag} = 0

C	1.50169	0.06170	-0.84808
C	2.88192	-0.16237	-1.51759
C	0.62797	-1.14033	-0.84843
N	-0.62026	-1.15800	-0.59557
H	1.11200	-2.09934	-1.04611
Al	-1.78414	0.28954	0.07670
Cl	-1.11561	2.19925	-0.54433
Cl	-1.58022	-0.06400	2.16074
Cl	-3.67882	-0.21092	-0.72085
H	0.96905	0.89301	-1.30443
H	3.01283	0.51860	-2.35783
H	3.01292	-1.17930	-1.88961
C	1.89538	0.48192	0.62333
C	2.53696	-0.73711	1.25639
C	3.71670	-0.91855	0.66523
C	3.88447	0.17264	-0.37404
C	3.13728	1.32871	0.31285
H	1.08069	0.94987	1.16856
H	2.05601	-1.37425	1.98594
H	4.40121	-1.74280	0.81087
H	4.89844	0.36326	-0.71428
H	2.92921	2.16984	-0.35038
H	3.63294	1.67263	1.21913
C	-1.30554	-2.45804	-0.58040
H	-2.05777	-2.47193	-1.36596
H	-0.59061	-3.26736	-0.72113
H	-1.81339	-2.57534	0.37716

P: exo_CP + MeN-AlCl$_3$
E = −2028.539510
H = −2028.523525
G = −2028.583525
N_{imag} = 0

C	3.83454	-1.08587	0.23115
C	2.91769	-1.32101	-0.70513
C	2.24896	-0.00228	-1.02508
C	3.79315	0.39310	0.55580
C	3.42134	0.96934	-0.81959
H	4.42783	-1.81736	0.76119
H	2.58976	-2.27540	-1.09076
H	1.68792	0.04309	-1.95431
H	4.66779	0.80552	1.05124
H	3.13084	2.02161	-0.78118
H	4.20382	0.83038	-1.56327
C	1.37551	0.29285	0.26277
C	2.45900	0.63540	1.32345

C	0.45903	1.42478	0.00293
N	-0.80734	1.34638	-0.11452
H	0.92054	2.40722	-0.12780
H	0.81592	-0.59562	0.53616
H	2.35519	0.01064	2.20781
H	2.39273	1.67852	1.64060
Al	-1.81532	-0.34209	0.05022
Cl	-1.48622	-0.95717	2.05047
Cl	-0.95295	-1.60344	-1.42469
Cl	-3.81199	0.21480	-0.36706
C	-1.56845	2.56363	-0.40772
H	-2.30835	2.71680	0.37616
H	-0.90365	3.42441	-0.47073
H	-2.10476	2.43185	-1.34587

RC: endo_CP + $N(C_4H_8)^+$

E = −522.243870

H = −522.229292

G = −522.286367

N_{imag} = 0

C	0.46668	1.68200	0.22333
C	1.59638	2.19461	-0.27653
H	2.26274	2.79401	0.32956
C	-0.37883	0.89646	-0.62769
N	-1.50166	0.37461	-0.28290
H	-0.07025	0.74763	-1.65846
H	0.18101	1.84422	1.25411
H	1.87458	2.04680	-1.31210
C	1.45917	-1.40355	0.51172
C	1.79739	-1.37803	-0.79094
C	3.02757	-0.60731	-0.95316
C	3.43521	-0.17195	0.25233
C	2.49741	-0.67302	1.30413
H	0.62733	-1.93737	0.95046
H	1.27741	-1.88086	-1.59555
H	3.53358	-0.44529	-1.89434
H	4.32937	0.39649	0.46224
H	2.09247	0.12085	1.93959
H	3.01468	-1.36329	1.98170
C	-2.35269	-0.42758	-1.19057
H	-2.37278	0.03133	-2.17641
H	-1.91536	-1.42630	-1.26364
C	-2.08573	0.43742	1.07581
H	-1.33088	0.14835	1.80656
H	-2.39441	1.46622	1.26875
C	-3.27493	-0.51790	1.00324
H	-2.95870	-1.52858	1.26438
H	-4.06796	-0.22447	1.68607
C	-3.69151	-0.45824	-0.46765
H	-4.24917	0.45654	-0.67265
H	-4.29877	-1.30595	-0.77420

RC: exo_CP + N(C_4H_8)$^+$
E = −522.242399
H = −522.228802
G = −522.284206
N_{imag} = 0

C	3.16253	0.71209	-0.72442
C	1.92104	1.48231	-0.69857
C	1.35654	1.37270	0.51699
C	3.35099	0.14047	0.47713
C	2.23889	0.53960	1.39551
H	3.82476	0.64218	-1.57533
H	1.55035	2.07404	-1.52454
H	0.46077	1.87410	0.85790
H	4.19361	-0.46473	0.77790
H	1.73207	-0.31537	1.85686
H	2.62428	1.13779	2.22983
C	0.52035	-1.47588	-0.76806
C	1.60293	-2.20577	-0.48013
C	-0.46875	-1.25398	0.24398
H	-0.34358	-1.74468	1.20616
N	-1.52141	-0.53082	0.10442
H	0.38776	-1.02994	-1.74409
H	1.74476	-2.64732	0.49990
H	2.36967	-2.39263	-1.21870
C	-2.53781	-0.32728	1.16370
H	-2.72991	-1.26876	1.67314
H	-2.13906	0.39735	1.87710
C	-1.85532	0.24815	-1.10937
H	-0.97938	0.82201	-1.41173
H	-2.11958	-0.45289	-1.90310
C	-3.04469	1.10020	-0.67429
H	-3.69736	1.33611	-1.51074
H	-2.69383	2.03825	-0.24188
C	-3.72223	0.24394	0.39727
H	-4.29658	-0.56206	-0.06131
H	-4.38563	0.81300	1.04340

TS: endo_CP + N(C_4H_8)$^+$
E = −522.229800
H = −522.216823
G = −522.268891
N_{imag} = 1,-353.4007i cm^{-1}

C	-0.58748	-0.93670	-0.64710
C	-1.86742	-0.89909	-1.33471
H	-2.23281	-1.90132	-1.59317
C	0.36366	0.05268	-0.81644
N	1.58821	0.05604	-0.32892
H	0.11003	0.92265	-1.41794
H	-0.33152	-1.81371	-0.06774
H	-1.87892	-0.28221	-2.23287
C	-1.76458	0.37004	1.40248

C	-2.07368	1.48690	0.63882
C	-2.97247	1.10134	-0.33859
C	-3.13189	-0.33199	-0.32560
C	-2.71026	-0.72540	1.08663
H	-1.05751	0.34786	2.22415
H	-1.64418	2.47038	0.75365
H	-3.39304	1.75110	-1.09497
H	-4.05882	-0.73756	-0.73125
H	-2.32396	-1.73242	1.21574
H	-3.58363	-0.60655	1.74650
C	2.56201	1.14064	-0.52993
H	2.48662	1.53028	-1.54433
H	2.36402	1.95023	0.17743
C	2.14113	-1.02933	0.48371
H	1.48089	-1.23667	1.32979
H	2.20777	-1.93407	-0.12528
C	3.51504	-0.50648	0.90474
H	3.43673	0.03378	1.84903
H	4.23356	-1.31159	1.03543
C	3.88835	0.45853	-0.22295
H	4.23353	-0.09326	-1.09925
H	4.66145	1.16836	0.06040

TS: exo_CP + N(C_4H_8)$^+$

E = −522.229465

H = −522.216520

G = −522.268362

N_{imag} = 1,−338.7877i cm^{-1}

C	3.34260	0.66221	-0.51914
C	2.48550	1.61501	0.00380
C	1.75212	1.02209	1.01692
C	3.03857	-0.63776	0.03236
C	2.33449	-0.30059	1.34643
H	4.04663	0.82871	-1.32641
H	2.37248	2.62912	-0.35283
H	0.96553	1.50084	1.58562
H	3.85103	-1.37044	0.05124
H	1.63099	-1.03992	1.72214
H	3.10605	-0.13927	2.11330
C	0.58595	-0.54953	-0.90710
C	1.83957	-1.29129	-0.95411
C	-0.45731	-0.94528	-0.09714
H	-0.32809	-1.81366	0.54298
N	-1.64799	-0.37460	-0.01782
H	0.43888	0.27600	-1.58854
H	1.74411	-2.31001	-0.55512
H	2.27747	-1.35385	-1.95549
C	-2.73651	-0.83800	0.85536
H	-2.77502	-1.92573	0.86788
H	-2.56969	-0.47692	1.87634
C	-2.03629	0.79718	-0.80206

H	-1.29480	1.59211	-0.67518
H	-2.06789	0.53169	-1.86099
C	-3.41553	1.16092	-0.25250
H	-4.04315	1.63080	-1.00541
H	-3.31460	1.85345	0.58421
C	-3.96359	-0.18090	0.23859
H	-4.31998	-0.77856	-0.60265
H	-4.77798	-0.07551	0.95097

P: endo_CP + N(C_4H_8)$^+$

E = −522.266951

H = −522.255051

G = −522.305002

N_{imag} = 0

C	-0.73479	-0.54041	0.00079
C	-1.57727	-0.50315	-1.32257
H	-1.48846	-1.45955	-1.83584
C	0.34077	0.45728	-0.02745
N	1.59427	0.22095	0.04909
H	0.05330	1.50164	-0.13701
H	-0.33561	-1.53802	0.17046
H	-1.25654	0.27811	-2.01158
C	-1.82965	-0.22441	1.10236
C	-2.33558	1.18151	0.85609
C	-3.04395	1.15857	-0.27127
C	-3.02272	-0.25985	-0.80017
C	-2.98725	-1.05063	0.51637
H	-1.49885	-0.46043	2.11001
H	-2.09725	2.04349	1.46370
H	-3.49619	1.99992	-0.77724
H	-3.79209	-0.51913	-1.52110
H	-2.74934	-2.10735	0.38542
H	-3.89796	-0.93994	1.10071
C	2.64314	1.27336	-0.01348
H	2.33123	2.05565	-0.70081
H	2.75392	1.69030	0.98885
C	2.21774	-1.12570	0.16642
H	1.78591	-1.65520	1.01255
H	1.99589	-1.67374	-0.75007
C	3.70770	-0.82808	0.31343
H	3.96397	-0.70810	1.36654
H	4.31628	-1.63157	-0.09321
C	3.88352	0.49766	-0.42875
H	3.88530	0.33922	-1.50810
H	4.79662	1.02119	-0.15746

P: exo_CP + N(C_4H_8)$^+$

E = −522.265441

H = −522.253504

G = −522.303862

N_{imag} = 0

C	3.49283	0.76681	-0.32488
C	2.67008	1.36600	0.53323
C	1.65229	0.33760	0.97440
C	3.03836	-0.66825	-0.47623
C	2.49773	-0.94336	0.93490
H	4.29145	1.22425	-0.89094
H	2.64458	2.41034	0.80854
H	1.09401	0.56002	1.88059
H	3.76447	-1.36732	-0.87941
H	1.92477	-1.87034	1.00773
H	3.27540	-0.93563	1.69516
C	0.73758	0.13502	-0.32067
C	1.70593	-0.62195	-1.28152
C	-0.43780	-0.67283	0.02828
H	-0.28530	-1.70894	0.32495
N	-1.64868	-0.26315	0.05274
H	0.43470	1.10045	-0.71693
H	1.35495	-1.63485	-1.48809
H	1.79986	-0.10119	-2.23159
C	-2.80759	-1.11362	0.43469
H	-2.65216	-2.12483	0.06707
H	-2.86500	-1.12550	1.52437
C	-2.10681	1.10379	-0.31623
H	-1.53363	1.84200	0.24029
H	-1.92389	1.23877	-1.38306
C	-3.59682	1.09035	0.01381
H	-4.15290	1.76909	-0.62757
H	-3.75472	1.39522	1.04885
C	-3.99061	-0.37567	-0.17280
H	-4.08314	-0.61895	-1.23219
H	-4.92374	-0.63388	0.32117

RC: endo_CP + N(C_4H_8O)$^+$

E = −597.442483

H = −597.427228

G = −597.485689

N_{imag} = 0

C	0.67910	-1.58949	0.31686
C	1.73343	-1.95234	1.05710
H	2.37904	-2.76826	0.76000
C	-0.13112	-0.49565	0.76423
N	-1.20780	-0.06985	0.19758
H	0.17260	0.01707	1.67140
H	0.44732	-2.09029	-0.61281
H	1.96517	-1.45979	1.99263
C	1.96044	1.07999	-1.02840

C	2.18790	1.54351	0.21505
C	3.32416	0.83144	0.79339
C	3.78668	-0.05877	-0.10302
C	2.98888	0.04632	-1.36371
H	1.22218	1.44806	-1.72729
H	1.65317	2.34742	0.70355
H	3.73581	1.02055	1.77473
H	4.64000	-0.70976	0.01906
H	2.56822	-0.90766	-1.69607
H	3.62244	0.39600	-2.18818
C	-1.94209	1.09798	0.69438
H	-1.48007	1.44573	1.61524
H	-1.83627	1.88212	-0.05775
C	-1.74662	-0.66582	-1.04245
H	-1.10078	-0.38523	-1.87898
H	-1.73623	-1.74764	-0.92176
C	-3.18565	-0.19122	-1.22958
H	-3.23035	0.82257	-1.64161
H	-3.69806	-0.85700	-1.92034
C	-3.41779	0.72969	0.91303
H	-3.56852	0.32749	1.91343
H	-4.02480	1.63310	0.80069
O	-3.83752	-0.27241	0.01121

RC: exo_CP + N(C_4H_8O)$^+$
E = −597.440893
H = −597.425592
G = −597.484017
N_{imag} = 0

C	3.48685	0.72473	-0.71295
C	2.31123	1.54286	-0.42625
C	1.83980	1.23005	0.79427
C	3.72939	-0.08010	0.33580
C	2.72796	0.19482	1.41324
H	4.07158	0.78525	-1.61953
H	1.92086	2.30562	-1.08617
H	1.01663	1.70754	1.30809
H	4.54751	-0.77816	0.43669
H	2.20050	-0.70290	1.75425
H	3.22311	0.59976	2.30401
C	0.72469	-1.25492	-0.96627
C	1.74612	-2.10820	-0.83232
C	-0.20043	-1.10006	0.11473
H	-0.04914	-1.70422	1.00492
N	-1.21870	-0.31022	0.12863
H	0.61339	-0.65752	-1.85972
H	1.87815	-2.69951	0.06681
H	2.46822	-2.25044	-1.62401
C	-2.09328	-0.17598	1.29857
H	-1.79082	-0.89428	2.05682
H	-1.93761	0.82833	1.69657

C	-1.54282	0.58636	-1.00089
H	-0.78976	1.37874	-1.03960
H	-1.49752	-0.00303	-1.91507
C	-2.96024	1.11914	-0.81532
H	-3.32633	1.50644	-1.76346
H	-2.99986	1.93112	-0.08150
C	-3.55676	-0.38700	0.88194
H	-3.81497	-1.44434	0.91169
H	-4.19986	0.15282	1.58351
O	-3.78476	0.04504	-0.44276

TS: endo_CP + N(C_4H_8O)$^+$

E = −597.429749

H = −597.416021

G = −597.470045

N_{imag} = 1,-341.5490i cm^{-1}

C	0.80251	-0.71071	0.88713
C	1.95220	-0.45064	1.65532
H	2.40384	-1.29456	2.16261
C	-0.11342	0.30164	0.61427
N	-1.29085	0.16203	0.05096
H	0.14096	1.31655	0.90864
H	0.59856	-1.71959	0.55860
H	1.97822	0.45669	2.24435
C	2.23086	-0.08822	-1.45968
C	2.46158	1.19206	-1.03058
C	3.29134	1.13950	0.10646
C	3.50344	-0.19122	0.47935
C	3.10255	-1.02986	-0.70905
H	1.61935	-0.37239	-2.30495
H	2.05715	2.09131	-1.47125
H	3.64718	1.99818	0.66008
H	4.31104	-0.47779	1.13906
H	2.67966	-2.00922	-0.50282
H	4.00805	-1.18733	-1.31258
C	-2.16351	1.29498	-0.24336
H	-1.72147	2.20221	0.16304
H	-2.22185	1.40787	-1.32812
C	-1.79183	-1.15326	-0.36142
H	-1.21997	-1.51544	-1.22256
H	-1.65012	-1.84305	0.47176
C	-3.28057	-1.03981	-0.67175
H	-3.45695	-0.57382	-1.64754
H	-3.72615	-2.03194	-0.68976
C	-3.55648	1.05677	0.35621
H	-3.58742	1.38556	1.39415
H	-4.29119	1.63162	-0.21611
O	-3.89563	-0.31579	0.36242

TS: exo_CP + N(C_4H_8O)$^+$
E = –597.429347
H = –597.415582
G = –597.469762
N_{imag} = 1,-326.3548i cm^{-1}

C	3.68147	0.68685	-0.44720
C	2.88380	1.51006	0.37968
C	2.22142	0.71631	1.27216
C	3.44251	-0.65510	-0.15404
C	2.72831	-0.67837	1.17180
H	4.32639	1.04062	-1.24047
H	2.79630	2.58285	0.29504
H	1.52489	1.05459	2.02678
H	4.11328	-1.44413	-0.46643
H	1.99016	-1.46269	1.31975
H	3.49517	-0.80932	1.94823
C	0.79880	-0.25759	-1.02374
C	1.92126	-1.01589	-1.38936
C	-0.20044	-0.80767	-0.22758
H	-0.06115	-1.81404	0.15852
N	-1.34445	-0.24485	0.08876
H	0.67897	0.74075	-1.41846
H	1.90291	-2.07825	-1.17014
H	2.42184	-0.77681	-2.31699
C	-2.33032	-0.89205	0.95055
H	-2.00340	-1.90627	1.16998
H	-2.37327	-0.34262	1.89334
C	-1.69148	1.10038	-0.38246
H	-1.04290	1.83943	0.10018
H	-1.52349	1.13451	-1.45999
C	-3.16686	1.35826	-0.10068
H	-3.50292	2.21744	-0.67697
H	-3.34655	1.57303	0.95848
C	-3.70311	-0.90567	0.26608
H	-3.78921	-1.76335	-0.40003
H	-4.47926	-0.97892	1.03406
O	-3.90359	0.24374	-0.53344

P: endo_CP + N(C_4H_8O)$^+$
E = –597.465987
H = –597.453349
G = –597.505485
N_{imag} = 0

C	-1.01663	-0.49224	0.42303
C	-1.47019	-0.78996	-1.05694
H	-1.29025	-1.83887	-1.28987
C	0.07437	0.49120	0.43694
N	1.32454	0.23086	0.54109
H	-0.18707	1.54103	0.32263
H	-0.70970	-1.41420	0.91356
H	-0.94387	-0.18506	-1.79496

C	-2.34549	0.03619	1.08464
C	-2.71118	1.33697	0.39983
C	-3.09037	1.04052	-0.84223
C	-2.98990	-0.46051	-1.00836
C	-3.34028	-0.92692	0.41301
H	-2.30964	0.04455	2.17047
H	-2.61774	2.32068	0.83875
H	-3.35741	1.73255	-1.62844
H	-3.54862	-0.89459	-1.83218
H	-3.11969	-1.98019	0.59354
H	-4.36881	-0.70681	0.68891
C	2.35307	1.28219	0.54076
H	1.87017	2.25544	0.50623
H	2.88816	1.19598	1.48689
C	1.86466	-1.14804	0.62169
H	1.78236	-1.50221	1.65118
H	1.25826	-1.77310	-0.02999
C	3.31048	-1.13220	0.12397
H	4.00701	-0.80754	0.90401
H	3.59542	-2.13551	-0.18442
C	3.29194	1.06997	-0.65883
H	2.91450	1.58812	-1.53869
H	4.27858	1.47102	-0.40812
O	3.37546	-0.29630	-1.00278

P: exo_CP + N(C_4H_8O)$^+$
E = −597.464273
H = −597.451591
G = −597.503649
N_{imag} = 0

C	3.70847	0.84035	-0.28533
C	3.30749	0.67399	0.97255
C	2.26647	-0.42757	0.97281
C	2.94965	-0.14761	-1.14580
C	2.79019	-1.31786	-0.16411
H	4.38520	1.59360	-0.66212
H	3.57837	1.26097	1.83801
H	2.05440	-0.89319	1.93240
H	3.37107	-0.36754	-2.12200
H	2.08608	-2.07803	-0.51137
H	3.73524	-1.79059	0.09257
C	1.01164	0.23876	0.28585
C	1.48125	0.36273	-1.21081
C	-0.17114	-0.63023	0.37700
H	-0.02200	-1.70695	0.32220
N	-1.38460	-0.24529	0.51993
H	0.80532	1.21361	0.72100
H	0.88905	-0.27126	-1.87393
H	1.39448	1.38946	-1.55978
C	-2.50825	-1.18855	0.62565
H	-2.12384	-2.20540	0.62749

H	-2.98501	-0.99847	1.58768
C	-1.78513	1.18297	0.54451
H	-1.62125	1.57973	1.54839
H	-1.15214	1.71059	-0.16519
C	-3.24760	1.28310	0.10849
H	-3.44817	2.29129	-0.24677
H	-3.93490	1.07236	0.93448
C	-3.48035	-0.94924	-0.54217
H	-3.20022	-1.55070	-1.40523
H	-4.48776	-1.23432	-0.22431
O	-3.44597	0.39710	-0.96310

RC: endo_CP + NMe_2^+

E = −444.856859
H = −444.842813
G = −444.8979.11
N_{imag} = 0

C	-0.35005	1.56702	-0.01191
C	0.66661	2.09917	-0.70154
H	1.28671	2.88053	-0.28235
C	-1.13026	0.53337	-0.62271
N	-2.16602	-0.03454	-0.10352
H	-0.84878	0.20968	-1.61923
H	-0.57680	1.89222	0.99416
H	0.88938	1.78671	-1.71357
C	1.07144	-1.23343	0.80262
C	1.29744	-1.44636	-0.50775
C	2.38780	-0.58403	-0.95384
C	2.82422	0.14684	0.08823
C	2.05626	-0.23020	1.31486
H	0.36606	-1.76324	1.42776
H	0.79277	-2.17194	-1.13196
H	2.79005	-0.56754	-1.95692
H	3.64375	0.85054	0.08155
H	1.59825	0.62363	1.82369
H	2.72116	-0.69513	2.05321
C	-2.87654	-1.11046	-0.79373
H	-2.43743	-1.27106	-1.77406
H	-2.79969	-2.02169	-0.20019
H	-3.92552	-0.83691	-0.89784
C	-2.62962	0.29420	1.24838
H	-3.54891	-0.24957	1.44131
H	-1.87191	-0.00344	1.97443
H	-2.82074	1.36228	1.32911

RC: exo_CP + NMe_2^+
E = −444.855267
H = −444.841312
G = −444.895545
N_{imag} = 0

C	-2.66898	-0.21969	-0.77886
C	-1.59070	-1.18757	-0.96095
C	-1.00929	-1.43267	0.22725
C	-2.74360	0.11758	0.52058
C	-1.72209	-0.65718	1.29221
H	-3.31171	0.14054	-1.56925
H	-1.34047	-1.65536	-1.90345
H	-0.22197	-2.14783	0.42292
H	-3.46110	0.78693	0.97231
H	-1.06772	-0.02602	1.90351
H	-2.21158	-1.33996	1.99721
C	0.30252	1.42802	-0.50205
C	-0.63514	2.24006	0.00068
C	1.25027	0.82406	0.38196
H	1.20813	1.08595	1.43506
N	2.18231	0.00320	0.03314
H	0.33769	1.20587	-1.55922
H	-0.68466	2.46306	1.06051
H	-1.36546	2.71871	-0.63636
C	3.12015	-0.55859	1.00525
H	2.92535	-0.14169	1.98903
H	2.99903	-1.64138	1.02907
H	4.13668	-0.31838	0.69641
C	2.31768	-0.45837	-1.35288
H	3.11136	-1.19732	-1.39585
H	1.37682	-0.90856	-1.67082
H	2.56844	0.37918	-2.00214

TS: endo_CP + NMe_2^+
E = −444.844504
H = −444.832058
G = −444.882141
N_{imag} = 1,−338.9114i cm^{-1}

C	0.15029	-1.10590	-0.35922
C	-1.00161	-1.26199	-1.15247
H	-1.48424	-2.23157	-1.13001
C	1.10198	-0.13910	-0.66952
N	2.27222	0.01783	-0.09383
H	0.88971	0.54904	-1.48289
H	0.32905	-1.78893	0.45883
H	-1.00771	-0.81259	-2.13687
C	-1.23489	0.73064	1.27782
C	-1.43158	1.58240	0.22337
C	-2.27562	0.94954	-0.71032
C	-2.53014	-0.36484	-0.30749

C	-2.13962	-0.44166	1.14769
H	-0.62332	0.93001	2.14707
H	-0.99637	2.56442	0.11137
H	-2.61303	1.38428	-1.64180
H	-3.35354	-0.93706	-0.71264
H	-1.74540	-1.38928	1.50433
H	-3.04262	-0.22355	1.73591
C	3.19830	1.06723	-0.49900
H	2.77651	1.62817	-1.32868
H	3.38275	1.74265	0.33726
H	4.14397	0.62364	-0.81043
C	2.67775	-0.84582	1.00650
H	3.67418	-0.56128	1.33084
H	1.98509	-0.73756	1.84421
H	2.69029	-1.88814	0.68624

TS: exo_CP + NMe_2^+

E = −444.844265

H = −444.831844

G = −444.881761

N_{imag} = 1,−319.9657i cm^{-1}

C	2.75719	0.38656	-0.54961
C	1.98501	1.49602	-0.13589
C	1.23067	1.12218	0.93944
C	2.41183	-0.73845	0.19794
C	1.64104	-0.23474	1.38978
H	3.45907	0.38961	-1.37288
H	1.97772	2.46479	-0.61258
H	0.52736	1.74913	1.47003
H	3.03862	-1.61901	0.24126
H	0.84304	-0.87058	1.76510
H	2.36232	-0.10788	2.20937
C	-0.15890	-0.56385	-0.92646
C	0.92522	-1.45453	-0.91912
C	-1.22455	-0.73509	-0.04931
H	-1.18586	-1.55545	0.66181
N	-2.32490	-0.01715	-0.01275
H	-0.19976	0.21877	-1.67014
H	0.82707	-2.36074	-0.33080
H	1.47948	-1.59504	-1.83633
C	-3.42060	-0.32674	0.89680
H	-3.16136	-1.19030	1.50342
H	-3.61468	0.52707	1.54648
H	-4.32238	-0.54880	0.32569
C	-2.49999	1.11579	-0.91101
H	-3.42237	1.62911	-0.65612
H	-1.66233	1.80776	-0.80301
H	-2.55314	0.77857	-1.94737

P: endo_CP + NMe_2^+
E = −444.879751
H = −444.868310
G = −444.916977
N_{imag} = 0

C	0.08338	-0.52292	-0.27048
C	0.82074	-0.84067	1.08434
H	0.73456	-1.90407	1.30488
C	-1.03682	0.39753	-0.04409
N	-2.28209	0.09433	0.00125
H	-0.79658	1.44450	0.12708
H	-0.25574	-1.44287	-0.74179
H	0.41628	-0.28626	1.93097
C	1.23846	0.10860	-1.14238
C	1.66348	1.40085	-0.47707
C	2.28481	1.08018	0.65670
C	2.28779	-0.42923	0.76881
C	2.38559	-0.82296	-0.71276
H	0.99730	0.15483	-2.20070
H	1.44141	2.39327	-0.84432
H	2.66248	1.75641	1.41063
H	3.01105	-0.86240	1.45323
H	2.18513	-1.87933	-0.89836
H	3.33197	-0.53602	-1.16498
C	-3.31735	1.11474	0.20614
H	-2.86208	2.09979	0.23923
H	-4.02596	1.05934	-0.61927
H	-3.83633	0.90755	1.14063
C	-2.78725	-1.27682	-0.16109
H	-2.07706	-1.98948	0.24536
H	-3.72742	-1.35561	0.37816
H	-2.96128	-1.47582	-1.21829

P: exo_CP + NMe_2^+
E = −444.878093
H = −444.866596
G = −444.915814
N_{imag} = 0

C	-1.01663	-0.49224	0.42303
C	-1.47019	-0.78996	-1.05694
H	-1.29025	-1.83887	-1.28987
C	0.07437	0.49120	0.43694
N	1.32454	0.23086	0.54109
H	-0.18707	1.54103	0.32263
H	-0.70970	-1.41420	0.91356
H	-0.94387	-0.18506	-1.79496
C	-2.34549	0.03619	1.08464
C	-2.71118	1.33697	0.39983
C	-3.09037	1.04052	-0.84223
C	-2.98990	-0.46051	-1.00836
C	-3.34028	-0.92692	0.41301

H	-2.30964	0.04455	2.17047
H	-2.61774	2.32068	0.83875
H	-3.35741	1.73255	-1.62844
H	-3.54862	-0.89459	-1.83218
H	-3.11969	-1.98019	0.59354
H	-4.36881	-0.70681	0.68891
C	2.35307	1.28219	0.54076
H	1.87017	2.25544	0.50623
H	2.88816	1.19598	1.48689
C	1.86466	-1.14804	0.62169
H	1.78236	-1.50221	1.65118
H	1.25826	-1.77310	-0.02999
C	3.31048	-1.13220	0.12397
H	4.00701	-0.80754	0.90401
H	3.59542	-2.13551	-0.18442
C	3.29194	1.06997	-0.65883
H	2.91450	1.58812	-1.53869
H	4.27858	1.47102	-0.40812
O	3.37546	-0.29630	-1.00278
